# Supplementary material for: Labels as a feature: Network homophily for systematically annotating human GPCR drug-target interactions
Source: Nat Commun. 2025 May 3;16:4121. doi: 10.1038/s41467-025-59418-6 (PMC12048553; doi:10.1038/s41467-025-59418-6)
Supplement: Supplementary file 1 — Supplementary Information [file 41467_2025_59418_MOESM1_ESM.pdf]

Supplementary Information for

Labels as a Feature: Network Homophily for Systematically Annotating human GPCR Drug-Target Interactions

Frederik G. Hansson[1†], Niklas Gesmar Madsen[1†], Lea G. Hansen[2], Tadas Jakočiūnas[1], Bettina Lengger[1], Jay D. Keasling[3,4,5,6], Michael K. Jensen[2], Carlos G. Acevedo-Rocha[1*], Emil D. Jensen[1*]

[1] The Novo Nordisk Foundation Center for Biosustainability, Technical University of Denmark, DK-2800 Kgs. Lyngby, Denmark

[2] Biomia Aps Lersø Parkallé 44, 3. tv., DK-2100 Copenhagen, Denmark

[3] Joint BioEnergy Institute, Emeryville, CA, USA

[4] Biological Systems and Engineering Division, Lawrence Berkeley National Laboratory, Berkeley, CA, USA

[5] Department of Chemical and Biomolecular Engineering, Department of Bioengineering, University of California, Berkeley, CA, USA

[*] Corresponding authors;
emdaje@biosustain.dtu.dk
cargac@biosustain.dtu.dk

[†] These authors contributed equally to the study

This PDF file includes:

supplementary Figures S1-S27
supplementary Tables S1-S12

The following supplementary material is provided with the manuscript.

Source Data for figures containing graphs.
S1 Data file of cost analysis of hGPCR-screens

Supplementary Notes

S2 Prior Work

The chemical homophily principle was developed together with Chemical space networks (CSNs), which allow for visualisation and interpretation of chemically related molecules. CSN's and similar methods have been developed to rationally engineer drugs by considering the chemical neighbourhood and building structure–activity relationship (SAR) (*1*), leading to canonical domain-specific language such as 'activity cliffs' (*2*). However, these methods are difficult to scale due to the quadratic dependence on number of compounds and arbitrary design choices in their construction and thus been decreasing in utility (*3*, *4*). Chemical similarity thresholds are essential in limiting the connectivity of CSNs to enable interpretation. (*3*). These network-based methods have found their way to established chemical bioinformatics companies (e.g. Chemaxon with Neo4j[1]) and have been key tools for SAR campaigns (*1*). Preliminary results on successfully utilising CSNs for lead-compound identification were recently demonstrated (*5*), using an edge propagation technique. Nevertheless, interpolation remains challenging, as the chemical space is insufficiently annotated and sparse.

Concurrently and analogously to CSNs, knowledge-graphs have emerged as expressive ways to mine and infer information from large relational-databases using dyadic interactions. Knowledge graphs are widely used across various fields: in search engines to improve search results, at the interface with machine learning models for knowledge retrieval (*6*), and in diverse biological applications (interactome like data) (*7*–*9*).

Recent work has begun reconciling knowledge graphs with graph neural networks that operate on them, for knowledge-aware inference in architectures termed: Knowledge Enhanced Graph Neural Networks (*10*, *11*). Our work formalises this framework in the DTI setting for hGPCR bioactivity prediction, building on previous work in other domains.

S3 A new paradigm: Labels as a Feature

An emerging paradigm known as training free graph neural networks (TFGNNs (Published: TMLR 8/2024) (*12*), show how labels as a features (LaF) is an admissible operation in a transductive node classification tasks: labels of neighbouring nodes are used to update the learned representations. The paradigm proves how this increases the expressive power over Graph Neural Networks (GNNs). Empirically the results show that even without training, prediction accuracy is incredibly high on common benchmark GNN datasets. Here, we demonstrate similar training free predictions that are on-par or outperform conventional trained compound-to-prediction architectures. Instead of framing the task as a transductive node classification task, we reframe it as a graph classification task with neighbourhood labels as edge features (Figure 1d) in a directed (transductive) graph.

S4 Explainable AI

Explainable AI (XAI) encompasses: Transparency, Justification, Informativeness, and Uncertainty estimation (*13*). The completely algorithmic predictions (argmax and mean value, taking the inductive bias of network homophily) are transparent (closed-form expression), justified (in relation to available data) but has low informativeness. A mean value prediction is not incredibly new nor useful information to target discovery or derivatization efforts. In the perspective of uncertainty estimation: while the class probabilities of the model do look calibrated (correlate with AUC-ROC and F1 scores), this is not a rigorous uncertainty quantification technique. In this respect, future work on neighourhood-to-prediction architectures could take great advantage of Gaussian processes (*14*–*16*) or Conformal prediction (*17*). The former works in smooth input spaces, which would likely work in homophilic networks, and the latter is a more flexible and rigorous uncertainty quantification technique which is underexplored in relation to black-box ML models for small-molecule property prediction (*18*). A natural extension to the method proposed are fully differentiable models, circumventing pre-computed compound representations and enabling feature-attributions, which is critical for explainable small molecule GNNs. Gradient-based feature attribution would then go back from prediction to the chemical neighbourhood (a list of compounds).

S5 Cost-efficient experimental chemical screening against GPCRs

When screening thousands of interactions, the price pr. well is a deciding factor on the total scope of a project. The calculation of these prices are, however not trivial, as there are many factors determining the total costs, such as salaries and differences in the laboratory requirements for handling mammalian cell lines, radioactive substances, or yeast. We chose to simplify this equation by only accounting for material costs associated with performing a screen of 4000 individual drug-GPCR interactions in vitro for all known

[1] `https://chemaxon.com/blog/presentation/neo4j_presentation`

methods available to us. Our combined calculations are available in supplementary File S2, and a summary of the available screen types and their associated advantages/disadvantages is available in supplementary table S2. This analysis identified yeast as the cheapest and most reliable option at 1$/well, where the cheapest alternative is Calcium staining assays on mammalian cell lines engineered to express the GPCR, for which we estimated a price of 1.8$/well.

S6 Identifying receptor independent effects

By normalising the raw reads of relative luminescence units with receptor-wise Z-score statistics, we were able to set a unified threshold of $|Z|>3$ (p-value < 0.003) for hit-determination, and the data could then be compared to prior art registered in our annotated dataset (Figure S23B and C). The distribution of hits across chemical space are available in Supplementary Figure S23A. Across all tested GPCRs, we observed 92% correspondence between prior art, consisting of 474 individual DTIs, which have a description in prior art and have been screened in this study (Supplementary Figure S23B). 381 of the 474 comparable DTIs are labelled with "no effect", and if only partial agonism and agonism is investigated, the correspondence drops to 68%. However, the majority of all discrepancies are caused by a single GPCR, as HTR1A performed exceptionally poorly, missing out on 19 out of 28 expected agonists and partial agonists, likely due to the reduced sensitivity and low dynamic range observed in the DRC (Supplementary Figure S21). When excluding HTR1A, the total correspondence rises to 89% for agonists and partial agonists, and to 95% including no effect DTIs. These results demonstrate the conservation of GPCR signalling in yeast across a variety of DTIs. The largest group of discrepancies were entries in ChEMBL and IUPHAR/BPS guide to pharmacology being classified as "no effect", which our screen identified as hits (supplementary figure S23). Hexachlorophene and chloroxine (known antifungals), also generally dampened the signal to a similar degree across all GPCRs (Except OPRM1 for chloroxine). Finally, bromocriptine displayed a strongly dampening effect on the CHRM3 GPCR only. By re-screening these compounds on the parental yeast strain ScFH237, we discovered that halogenated phenothiazines, low hydrophilic-lipophilic balance steroids, and all compounds carrying a 7-membered ring, dampened the luminescence signal independently from GPCR signalling (Supplementary Figure S24) and were consequently excluded from our downstream analyses.

S7 Other discrepancies

We observed cases where GPCR activation was measured despite prior art suggesting otherwise. For example, chloroxine activated OPRM1 (Z=8.8), and serotonin activated MTNR1A (Z=5.0) (Supplementary figure S24C). Both have previously been classified as having “no effect" on the respective GPCRs (CHEMBL1201862 (*19*), and CHEMBL1130532 (*20*), respectively). These could be explained by the comparatively high dynamic ranges on the yeast platform, which may have allowed it to detect DTIs that other screens might have missed. Chloroxine is known to bind OPRK1 (*21*), so based on phylogeny, it is not surprising that it also binds to OPRM1 as shown in our screen, despite the conflicting information from prior art. Activation of MTNR1A by serotonin has already been shown in other publications relying on yeast platforms (*22*, *23*), however, this information is not deposited on either ChEMBL or the IUPHAR/BPS guide to pharmacology, which is why an older assay, is the only reference on this DTI in the dataset (*21*). This demonstrates how the high dynamic ranges on the yeast platform (Supplementary Figure S21) can help catch DTIs, which other screening campaigns have missed. Finally, there is a total of 5 previously reported DTIs which the yeast based screen did not catch:

1. Nalbuphine on OPRM1
2. Tramadol on OPRM1,
3. Apomorphine on ADRA2A
4. Pilocarpine on CHRM3
5. Bethanechol on CHRM3

Nr. 2 can be explained, as tramadol is a prodrug, which is only active once oxidised by the human CYP2D6, which the yeast does not have, hence no activity is observed. The others are true mismatches, and could indicate a yeast bias on receptor signalling. In summary, our findings revealed 92 % correspondence between prior research in mammalian cells and the yeast platform, underscoring the viability of yeast as a cost-effective substitute for human cell lines (Supplementary Table SS6). HTR1A accounted for most discrepancies due to poor signalling in yeast, while other variations stemmed from compounds that reduced the reporter signal irrespective of GPCRs, indicating yeast-specific effects. By excluding GPCR-independent signal-dampening compounds and HTR1A, the quality of the gathered experimental dataset was drastically increased, and the correspondence to prior art was now 98%

S8 Chemical Space Network Construction in 20 Minutes

To realise CSNN, we first constructed CSNs from an adjacency matrix (Figure 2a), which encodes the graph structure connecting similar compounds (Figure 2b). We tackled two major challenges in large CSN utilisation:

(i) Accelerated CSN construction By using available tools to parallelize the calculation of Jaccard/Tanimoto similarity (*24*), we reduced the calculation of an all-vs-all CSN for 187 K compounds to 20 minutes on a home-computer (32.4 billion edges possible on a MacBook Pro 32 GB RAM, M1 Max @ 2.0 GHz x 10 cores). This makes the method accessible and can be done for any compound library. For moderately sized compound libraries (10 K), a CSN was established in 0.952 $\pm$ 0.002 seconds. In head-to-head comparison, published methods reported a 1 hour computation for 80 K compounds (*25*), our methods completed the same in 3.7 minutes, which was 16-fold faster than state-of-the art. The resulting computation was a sparse adjacency matrix which describes the connectivity of the graph (See Figure 1a) and forms the basis for querying and visualisation of data with CSNN.

(ii) Open-access CSN tools to Query Available Data We enabled the broader use of CSNs by developing a standalone user-friendly script, akin to (*25*), for visualising and utilising chemical neighbourhoods to understand the rapidly expanding datasets (Supplementary Figure S18), which are being procured. Our script queries the CSN and automates visual summaries of the neighbourhoods (Figure 1b). The visual neighbourhoods give an overview of available data and help infer possible DTIs of a given compound. These tools Querying of the hGPCR dataset and visual reporting was achieved in < 6 seconds, and an output .csv was produced with associated DTIs, ChEMBL ID, and compound information as well as Figure 1a,b.

S9 Supplementary Figures

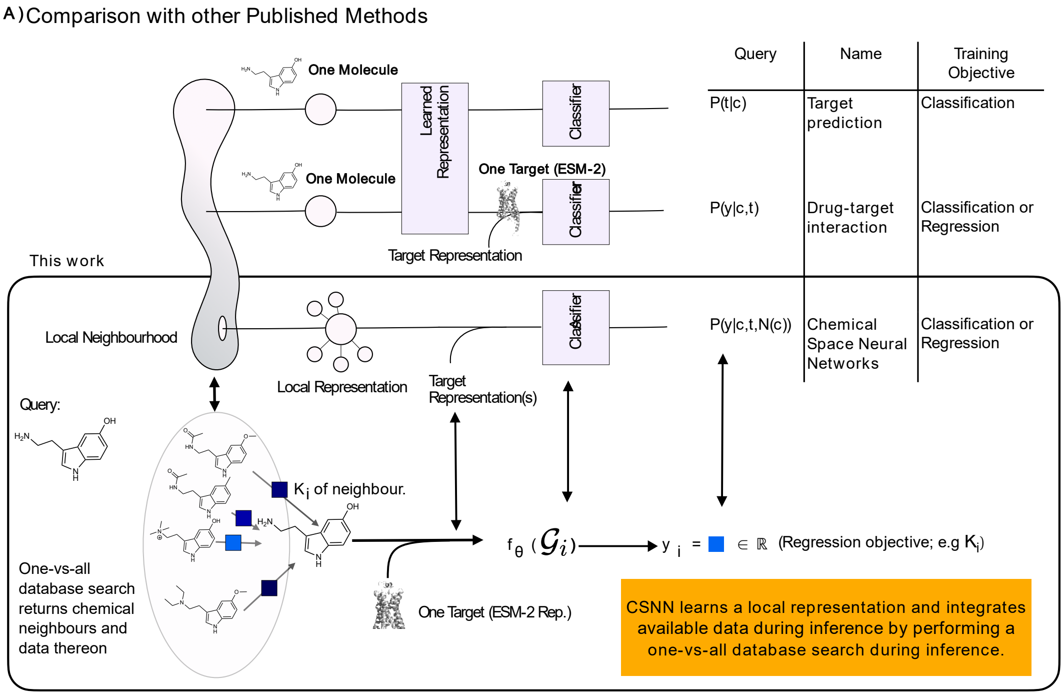

Figure S1: **Comparison of compound-to-prediction vs. neighbourhood-to-prediction architectures using LaFs**. Most drug-target interaction prediction methods indirectly either provide a conditional likelihood for the target given the compound P(t|c) or the conditional likelihood of activity y given the compound and target pair: P(y|t,c). These both take a *single* compound from chemical space (as visualised by the line) and use RDKit of MPNNs (like ChemProp) to produce a learned representation which is fed to a trained classifier. In the latter case, a target representation is concatenated to the compound representation. This work (bottom row), establises LaFs using neighbourhood-to-prediction architectures. An example for Serotonin is given to visually explain inference: the query is used in a one-vs-all database search which returns chemical neighbours and data thereon. This is a list of compounds and an accompanying list of labels (LaFs). Using a transductive node classification scheme, but re-framed as graph classification we can produce a single output by message-passing on the chemical neighbourhood graph. As stated in the main text, LaFs provide an approximate answer to guide ML model predictions.

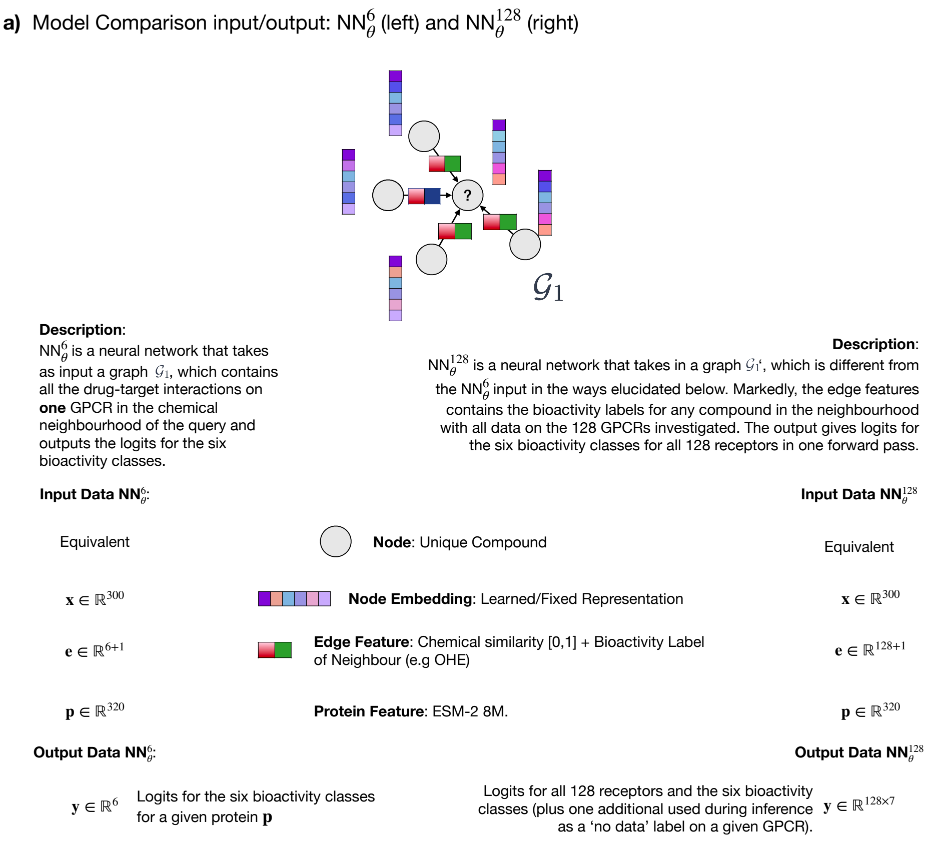

Figure S2: Comparison of the NN_θ^6 and NN_θ^{128} model used for drug-target interaction prediction (classification). The input and output parameters are compared explicitly, showing how the NN_θ^6 model requires one forward pass per potential DTI to product the six bioactivtiy class logits, while the NN_θ^{128} can predict logits for the six bioactivity class for all 128 GPCRs for a given compound in one forward pass.

a) NN$_\theta^6$ test metrics by class with only confident predictions.

		Precision	Recall	F1-Score	Support
**Threshold > 0.8**	**No Effect**	0,9713	0,9237	0,9469	4391
	**Allosteric binding**	0,9540	0,9706	0,9622	5234
	**Antagonist**	0,9534	0,9854	0,9691	4031
	**Inverse Agonist**	0,9153	0,8438	0,8780	64
	**Partial Agonist**	0,9085	0,7771	0,8377	166
	**Agonist**	0,9091	0,9701	0,9386	268
	Overall	0,9576	0,9574	0,9571	14154

b) NN$_\theta^6$ test ROC-AUC at optimal class thresholds.

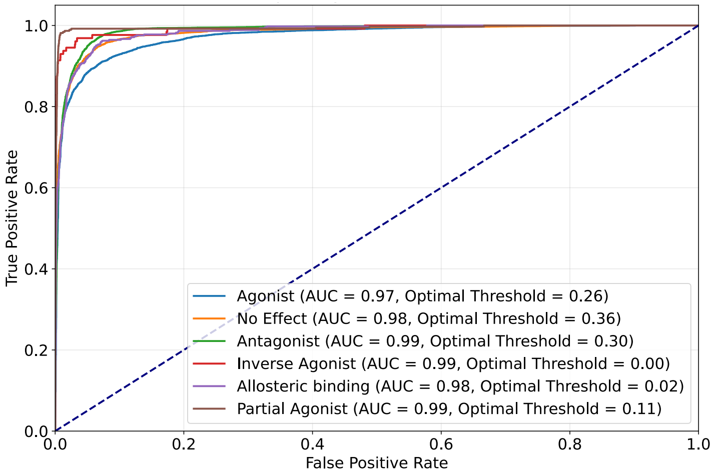

c) NN$_\theta^6$ test ROC-AUC non-optimised thresholds.

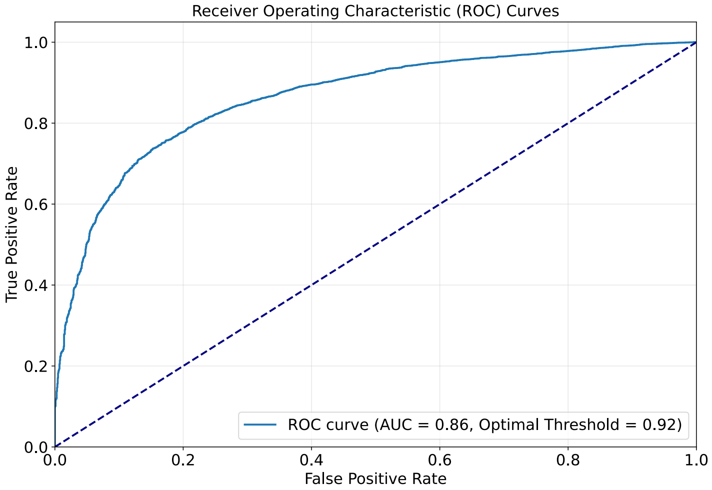

Figure S3: **NN$_\theta^6$ Test Metrics**. **(a)** Summarises the precision, recall, F1-score and statistical support for predictions with a class label probability > 0.8. Summaries are produces on a class-by-class basis. **(b)** ROC-AUC plots considering the class probability p_i and the true label $y_i \in 0, 1$. The AUC is provided for optimised probability thresholds. **(c)** Setting a single threshold for all classes significantly reduces model performance: AUC. Given results in Figure 2g, S4-S6 we suggest user to use a probability threshold of > 0.8 or to look at the entropy of the logits.

a) NN_{θ}^{6} Confusion matrix on test data unnormalised (raw counts, left) and normalised (row-wise, right) at varying logits probability.

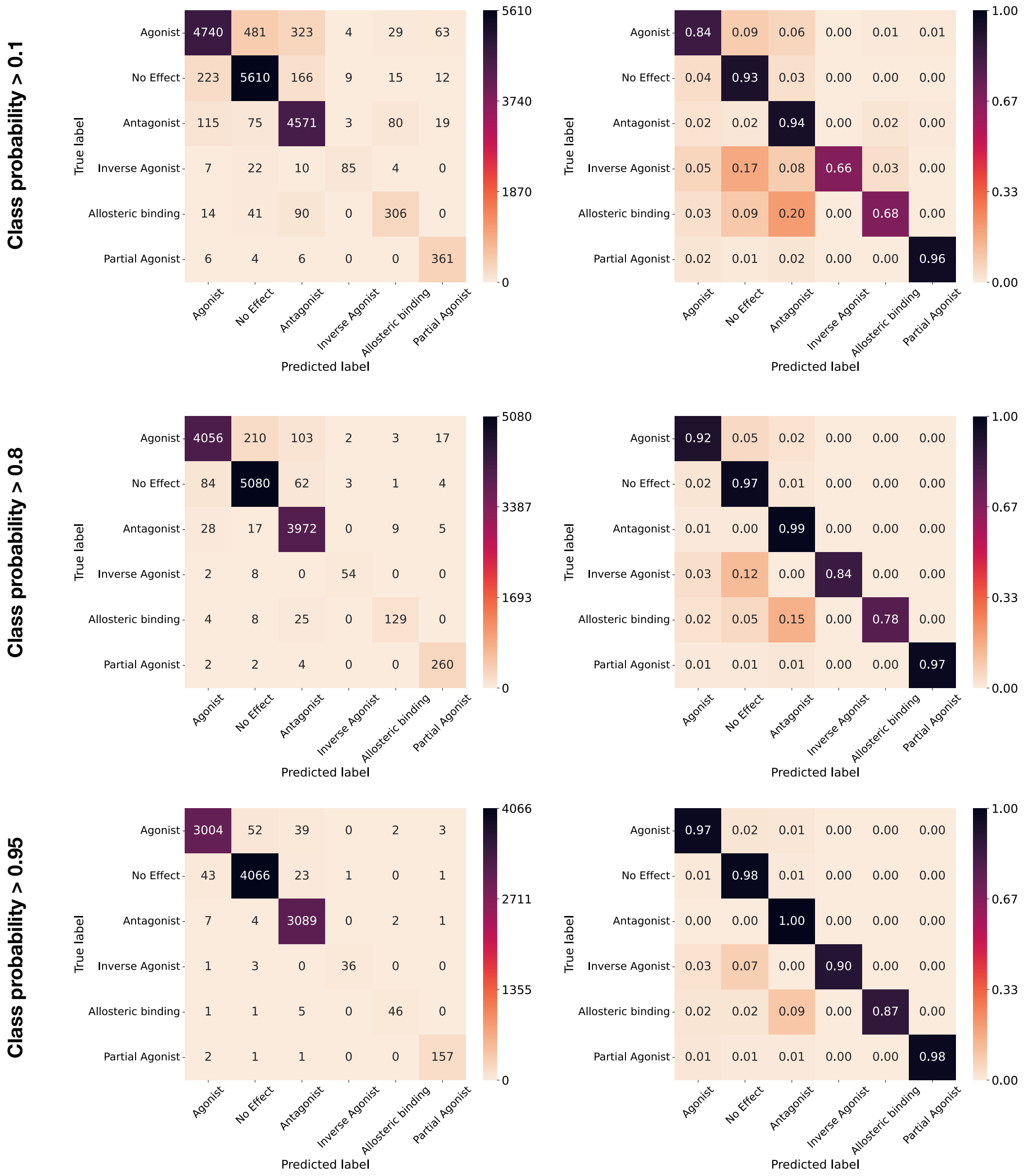

Figure S4: **Confusion Matrices for Filtered by Class Probability**. Un-normalised raw counts (left) and row-wise normalised confusion matrices (right). At a class probability of >0.8 the classification is excellent, even more so at 0.95 although with far less predictions passing the filter.

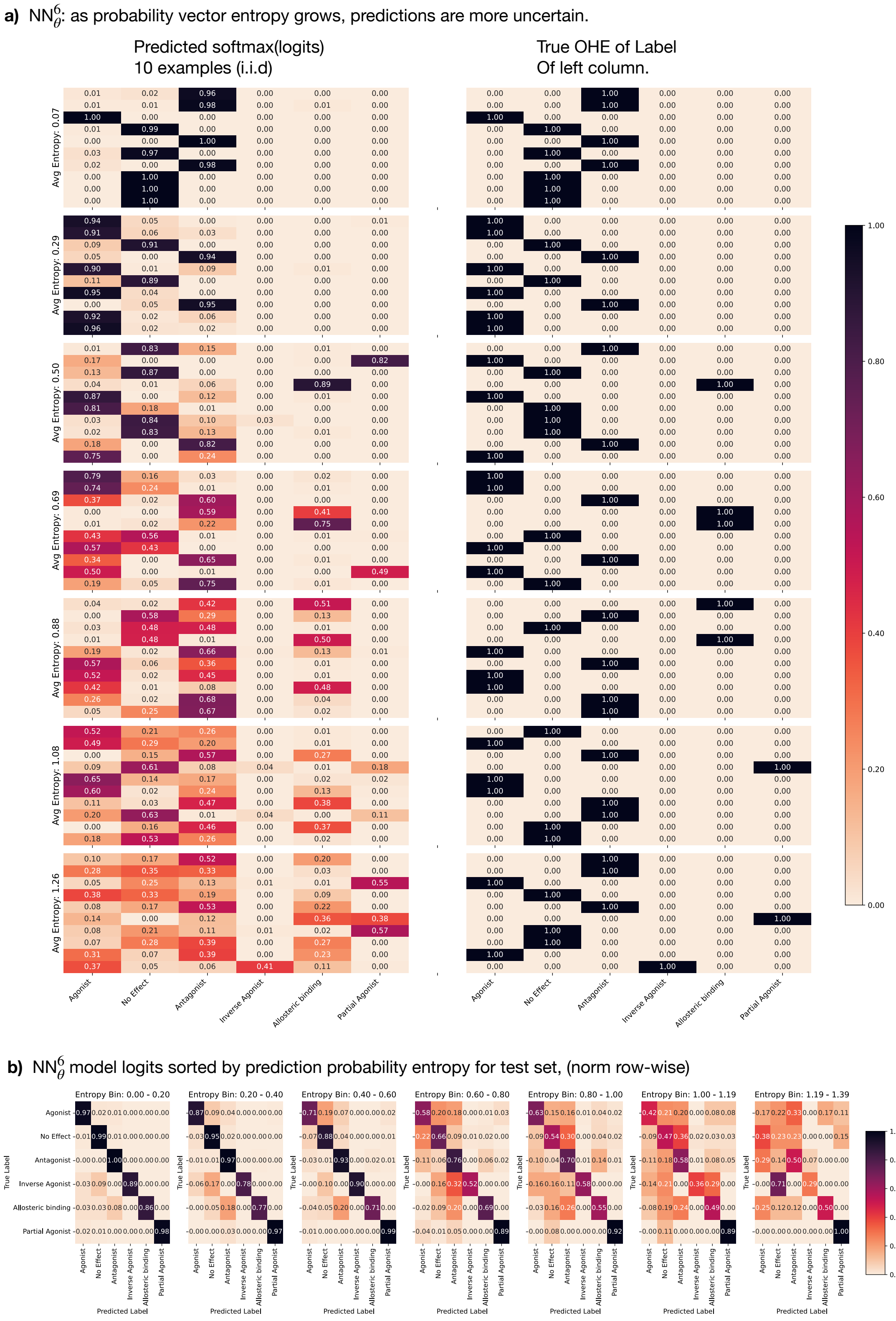

Figure S5: **Relating the Class Probability Entropy with the Predictive Performance of the Model**. **(a)** We calculate the class probability entropy (low if strongly peaked, high if dispersed) and sample 10 test examples i.i.d from each entropy bin. Top column shows that the one-hot encoding of the true labels (right) is strongly reflected in the predicted class labels (left, which are softmax(logits)). As the prediction entropy grows, the probability is spread across classes and looses it's correlation with the true one-hot encoding of the class label. **(b)** We compute confusion matrices on the test set, filtered by entropy bins to observe where probability is incorrectly assigned. For example, at 0.6-0.8 the antagonist and inverse agonist class is confused for 32% of predictions.

a) NN$_{\theta}^{6}$ model logits sorted by prediction probability entropy for test set (unnormalised)

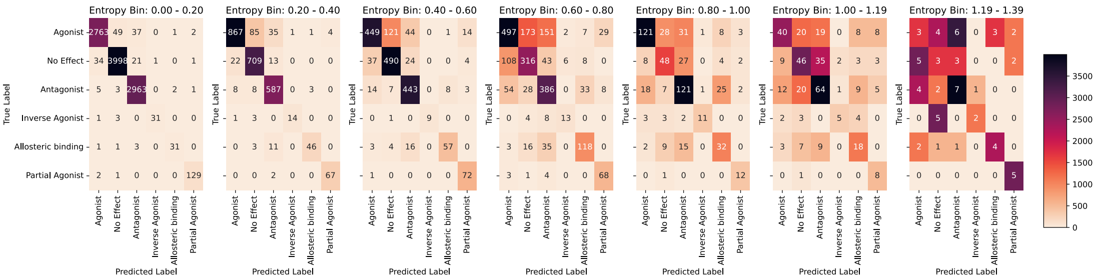

b) NN$_{\theta}^{6}$ predictions: AUC-ROC falls as model logits sorted by prediction probability entropy grow.

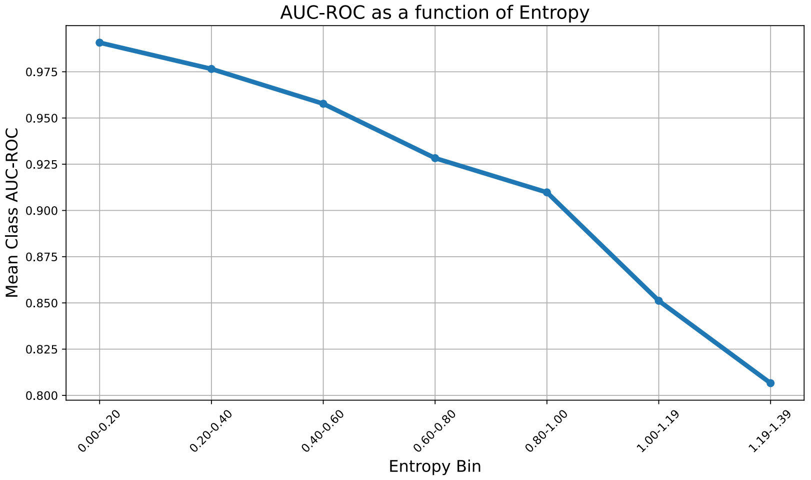

Figure S6: **Characterising the model performance filtering by prediction entropy shows well calibrated confidence**. **(a)** The same as Figure S5b but in unnormalised form. **(b)** The mean class AUC-ROC as a function of the sorted entropy bin shows a strong negative correlation between prediction entropy (non-peaked probability in class label) and the discriminative performance. Source data are provided in the Source Data file for panel b.

a) A single forward pass for all 128 receptors: NN_θ^{128}, test metrics breakdown by class

		Precision	Recall	F1-Score	Support
**Non-Threshold Predictions**	**No Effect**	0.8573	0.8265	0.8416	7221
	**Allosteric binding**	0.7514	0.7143	0.7324	385
	**Antagonist**	0.8552	0.8635	0.8593	6424
	**Inverse Agonist**	0.7632	0.4462	0.5631	130
	**Partial Agonist**	0.6905	0.3194	0.4367	454
	**Agonist**	0.8374	0.9201	0.8768	5205
**At optimal ROC-AUC Threshold**	**No Effect**	0.8123	0.8797	0.8446	7221
	**Allosteric binding**	0.3130	0.9351	0.4691	385
	**Antagonist**	0.8345	0.8812	0.8572	6424
	**Inverse Agonist**	0.0716	0.8538	0.1321	130
	**Partial Agonist**	0.1855	0.9009	0.3076	454
	**Agonist**	0.8006	0.9500	0.8690	5205

b) NN_θ^{128} ROC-AUC curve by class breakdown.

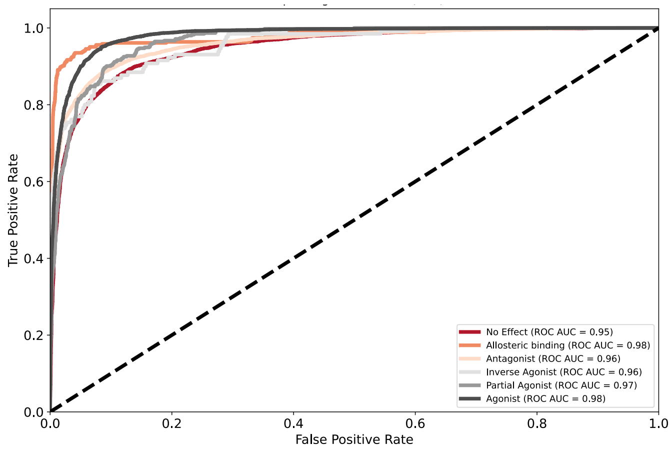

c) NN_θ^{128} performance is correlated with training class support.

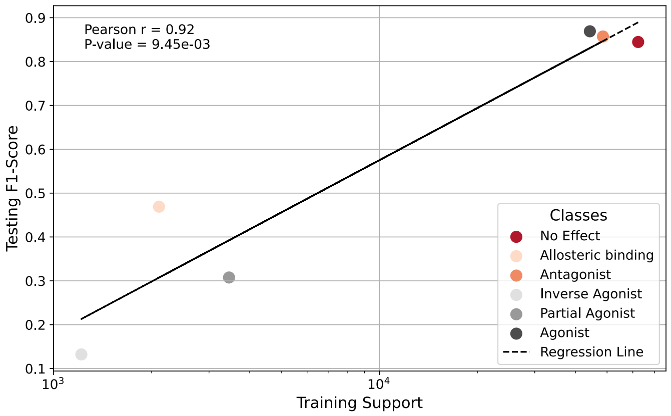

Figure S7: **Performance Metrics for the NN_θ^{128} model**. **(a)** Class-wise breakdown of precision, recall, and F1-score. Due to the class imbalance (low support), at optimal ROC-AUC the F1-score falls significantly for the low support classes and recall increases. **(b)** ROC-AUC plot using the probability p_i for the class label and the true label $y_i \in 0, 1$ shows strong descriminative performance. **(c) Testing F1-score correlates strongly with the training support. Source data are provided in the Source Data file for panels b and c.**

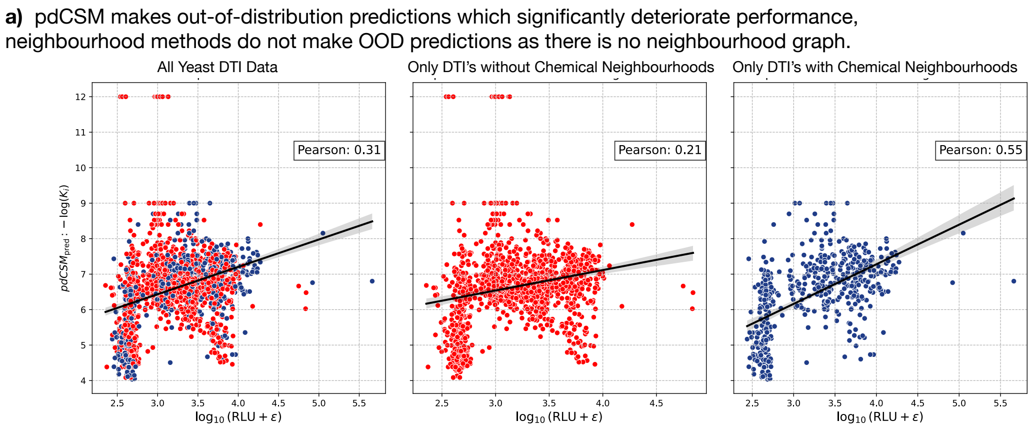

Figure S8: **pdCSM Bioactivity Predictions of Yeast Dataset Show OOD Behaviour**. For all investigated DTIs for which we have measured experimental RLU, we compare the correlation with the predicted K_i (left) which shows low Pearson correlation and many predictions at 12, indicating OOD behaviour. 12 corresponds to a K_i of picomolar, which is rare. Middle: Using our CSN we filtered for prediction for which there does not exists a chemical neighbourhood on a given DTI, this significantly reduces the correlation. Right: If we instead only pick DTIs for which there *do exist* chemical neighbourhoods, then the Pearson correlation markedly increases. This suggests that pdCSM makes out-of-distribution predictions that significantly deteriorate the performance of the model on unseen DTIs which are experimentally validated. Source data are provided in the Source Data file.

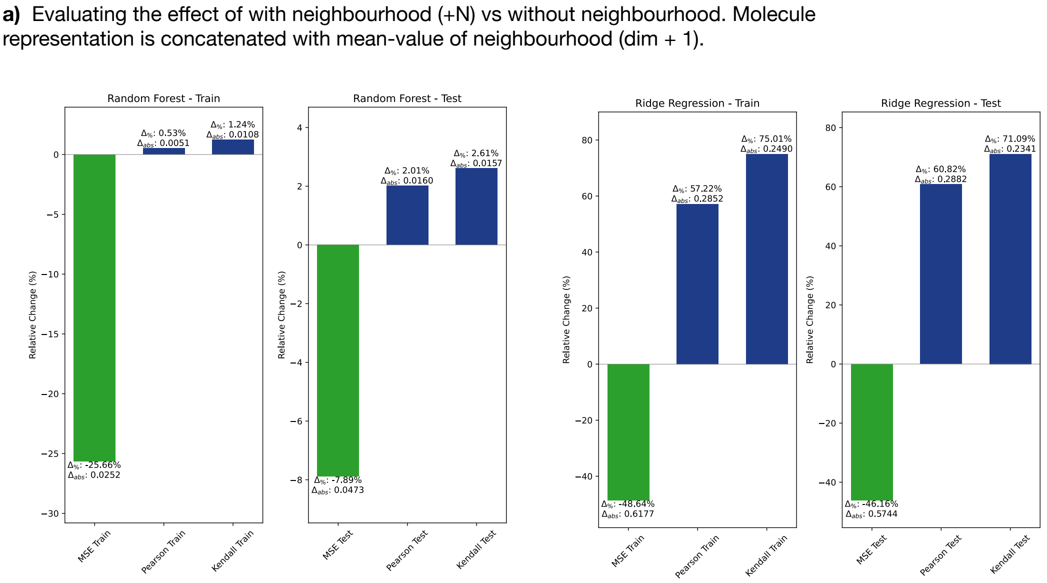

Figure S9: **Probing the LaF Effect**. Using two common ML models, we compare their relative performance on the training and testing test when *using* LaFs against not using LaFs. In the regression setting this corresponds to a change of input dimension by 1 (the mean value of the neighbourhood). For the higher-capacity RF model the increase in Pearson and Kendall correlation is smaller, but the inclusing of LaFs drive down the MSE significantly. In Figure 3d we provide a Table summarising the LaF effect on a low-capacity model like ridge regression. Here the results are illustrated, which shows that for both the training and testing set the MSE is reduced while the Pearson and Kendall correlation are increased significantly upon the inclusion of LaFs. Source data are provided in the Source Data file.

a) Graph Homophily: Mean-value predictions are good approximations. Per-receptor metrics reported.

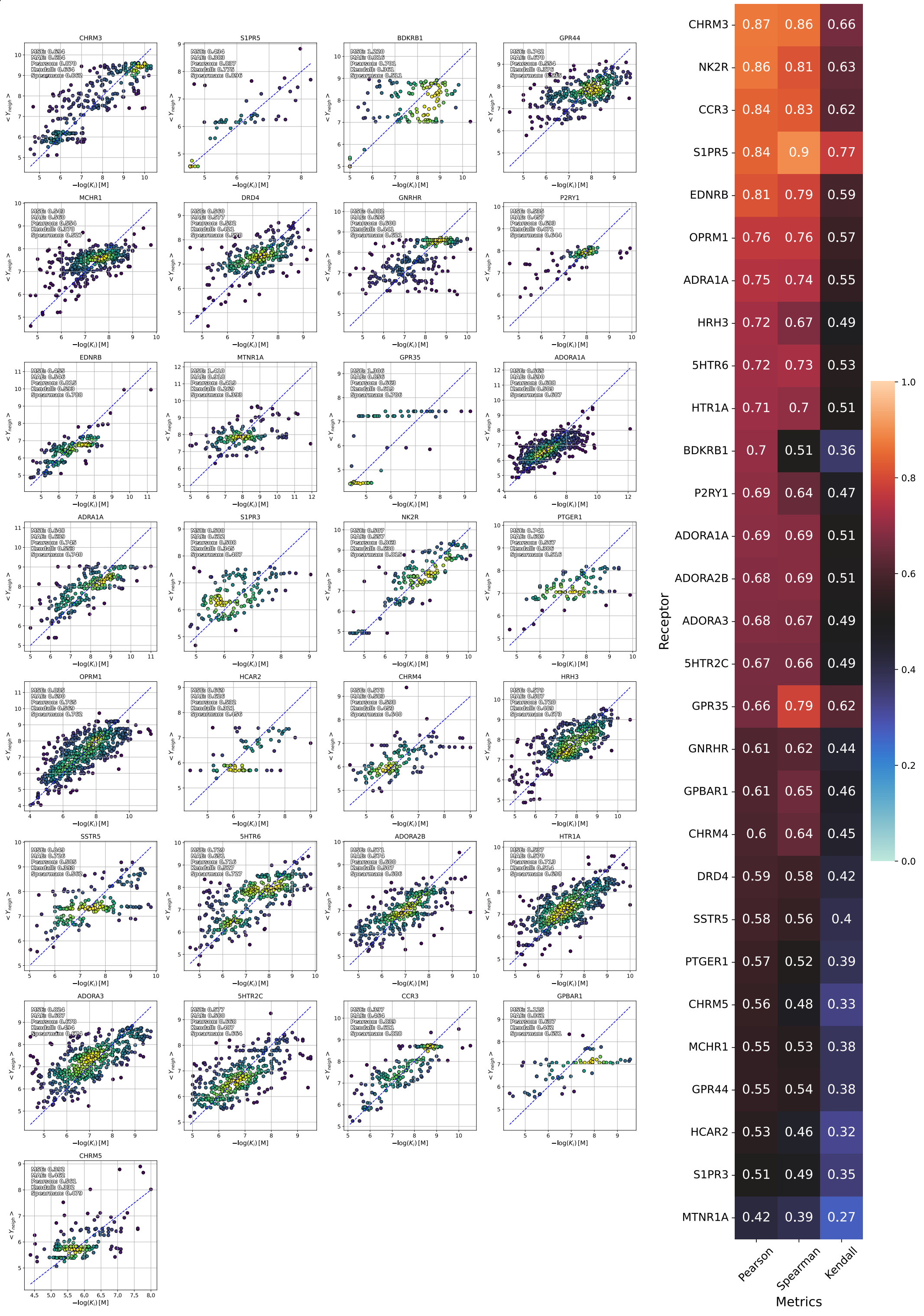

Figure S10: **Network Homophily as a Training Free Prediction**. If a network is homophilous, the mean value is a good approximation of the true value. In extension to Figure 3b,c,e and Table S1 we provide regression plots split by hGPCR to illustrate the performance of the mean value ($< Y_{neigh} >$ in detail). The heatmap (right) illustrates the Pearson, Spearman, and Kendall correlation coefficient under this training free prediction.

a) Graph Homophily: Comparing several aggregation techniques (mean, max, weighted, max similarity compound).

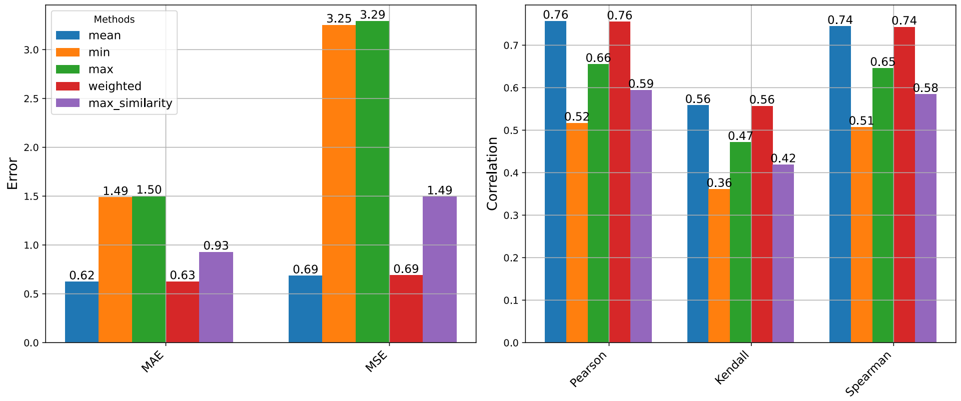

b) Graph Homophily: The test set regression plots excluding neighbourhoods by similarity.

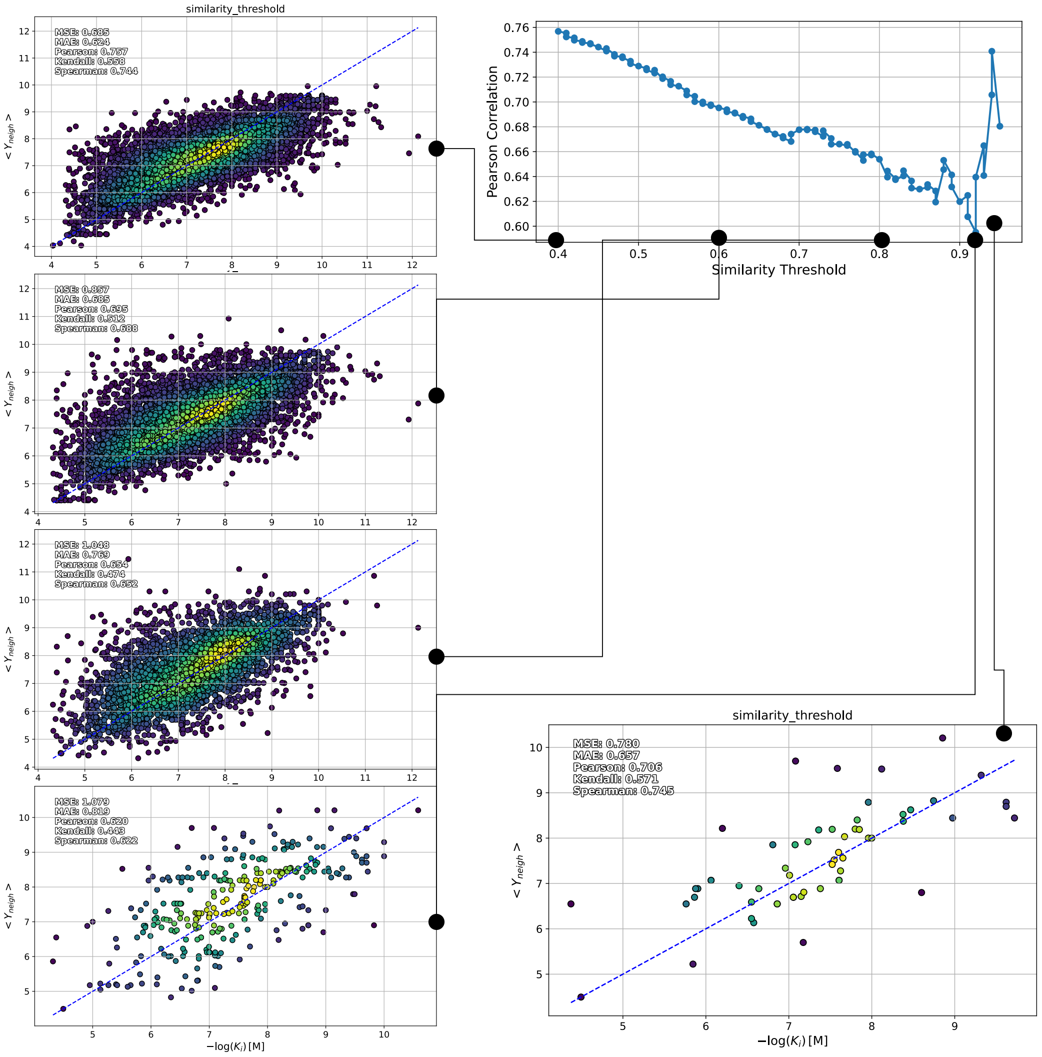

Figure S11: **Training Free Predictions, Aggregating Techniques**. **(a)** We find that the mean value offers a reliable training free prediction for the pdCSM test set. Min and max value, expectedly, are bad predictions. Surprisingly, taking only the most-similar molecule y-value (LaF), the prediction is also bad. **(b)** Pearson correlation filtered for the similarity threshold shows a striking negative correlation. Regression plots are shown at selected intervals, showing also the reduction in data-points. Curiously, keeping all neighbourhood data ensures the best prediction. Source data are provided in the Source Data file.

a) Train/Test metrics for MSE-loss objective on pdCSM data (CSNN). Fixed RDKit representation.

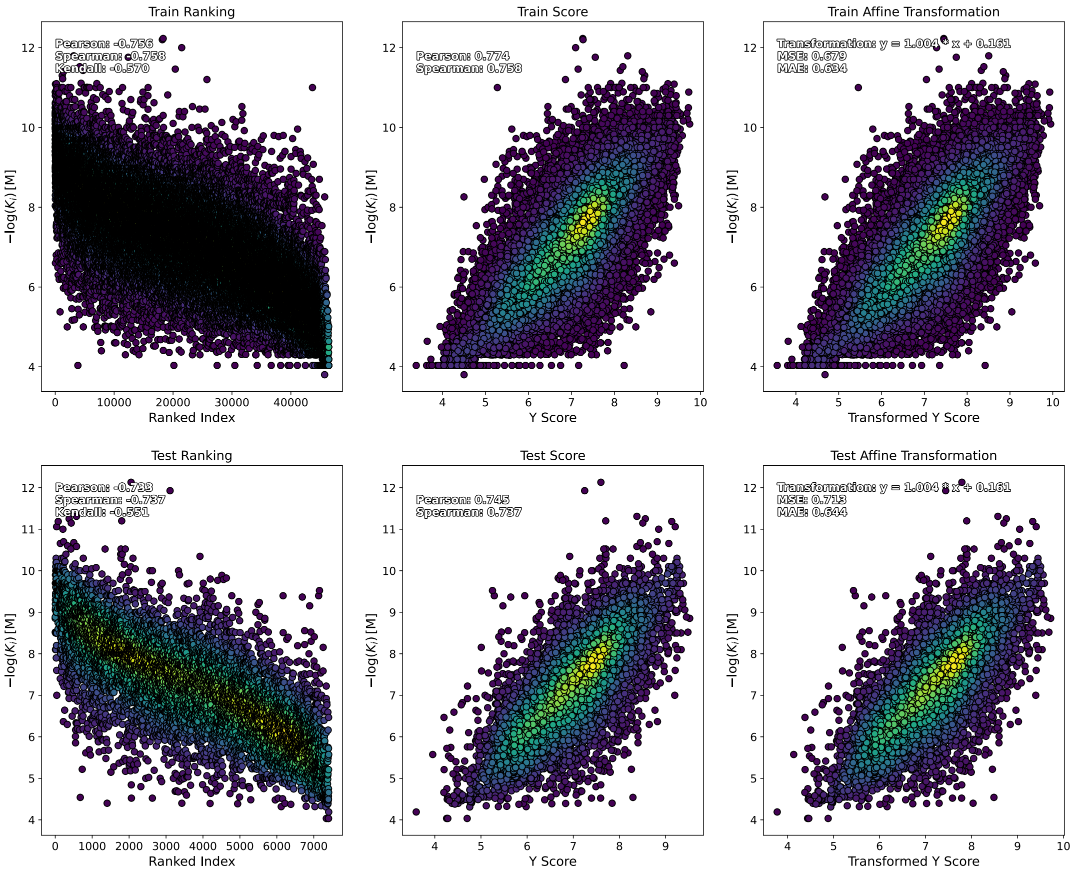

Figure S12: Train/Test metrics for the pdCSM dataset using the CSNN model, MSE-loss, and fixed RDkit compound representations (copying those from the original paper, without feature selection). Source data are provided in the Source Data file.

a) Training curves for BT-loss objective.

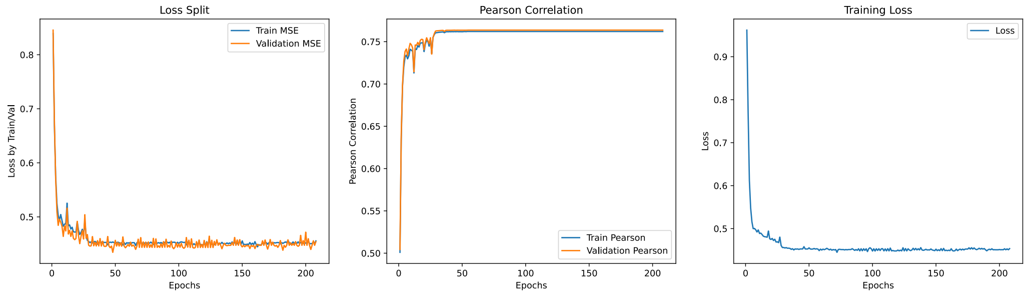

BT-loss - fixed

b) Train/Test metrics for BT-loss objective on pdCSM data (CSNN). Fixed RDKit representation.

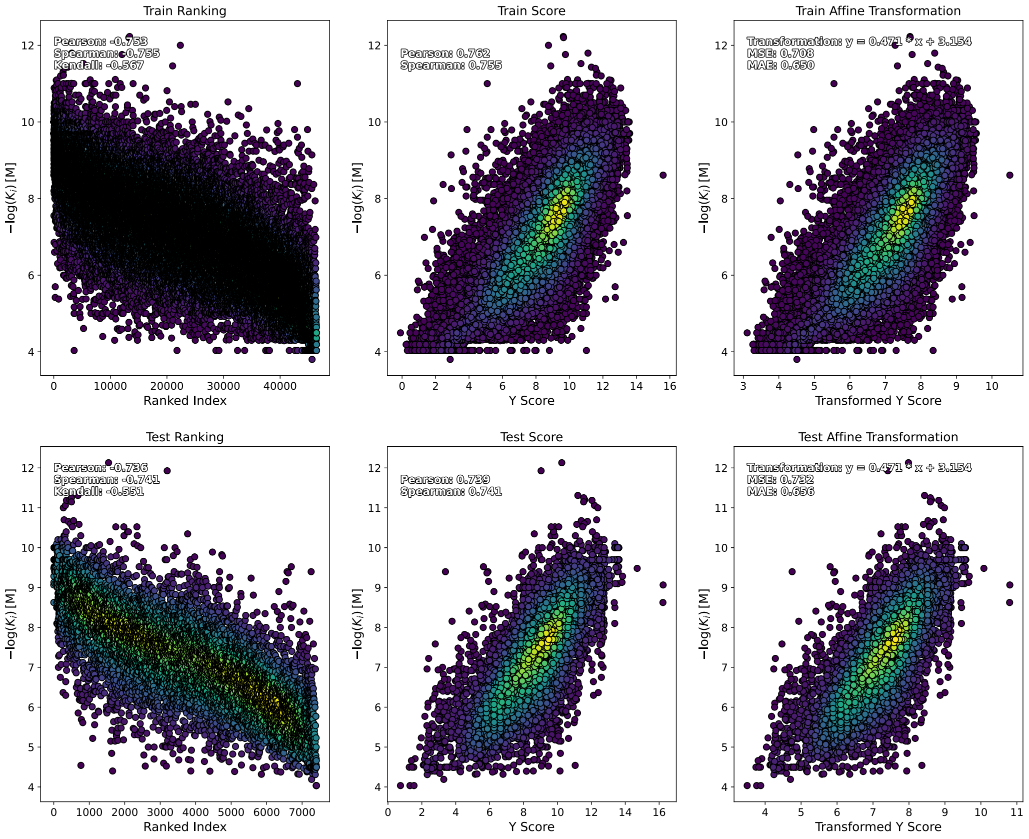

Figure S13: **a)** Training curves for BT-loss objective, showing, loss split, pearson correlation and training loss. **b)** Train/Test metrics for the pdCSM dataset using the CSNN model, a BT-loss (contrastive), and Train/Test metrics for the pdCSM dataset using the CSNN model, a BT-loss (contrastive), and fixed RDkit compound representations (copying those from the original paper, without feature selection). Due to the BT-loss not learning the true y-label an affine transformation is fit on the training data and used to align the predicted scores with the true scores for error metric calculation. The same affine transformation is performed on the test set. BT-loss models learn a ranking function rather than a regression objective, which up to an affine transformation, is equivalent to the true y-label. Source data are provided in the Source Data file.

a) Train/Test metrics for MSE-loss objective on pdCSM data (RF + N). Fixed RDKit representation.

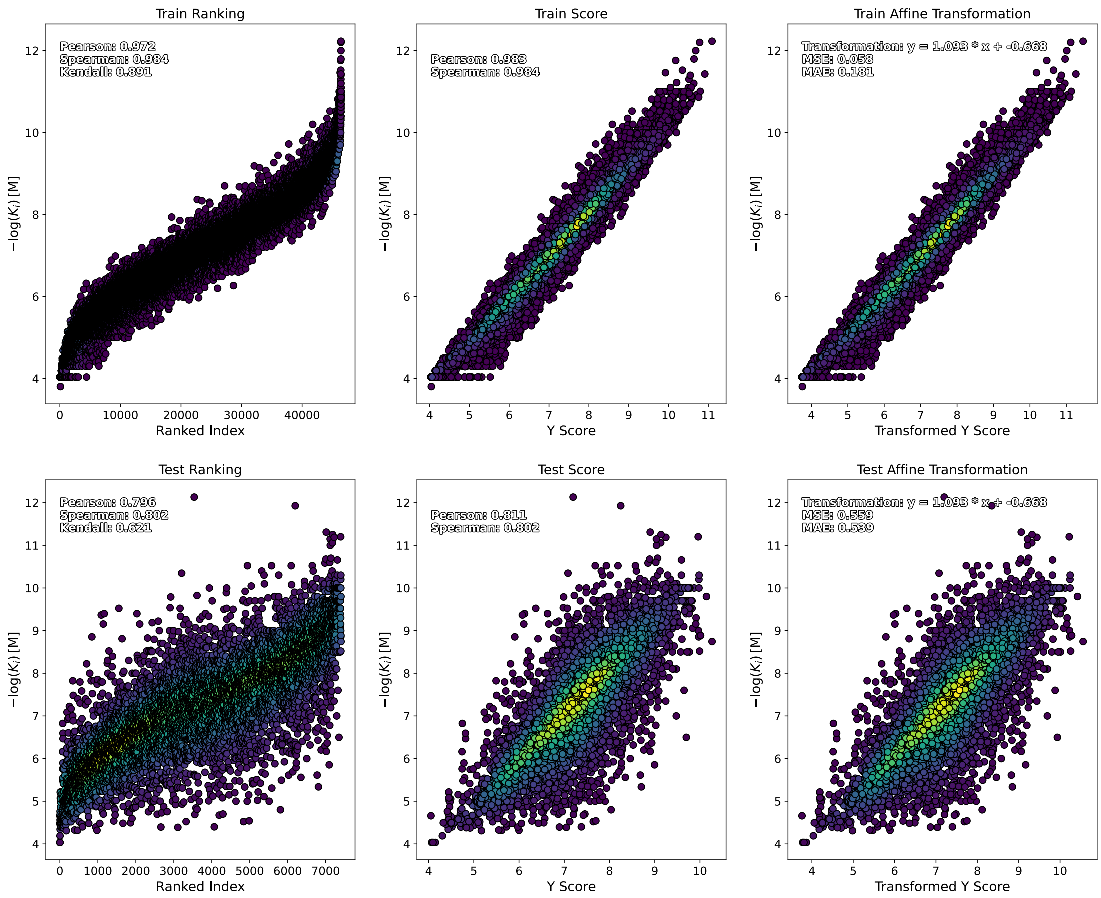

Figure S14: Train/Test metrics for the pdCSM dataset using the RF + N model, a MSE-loss, and fixed RDkit compound representations (copying those from the original paper, without feature selection). Interestingly, Figure S12, S13 show no signs of over-fitting for the CSNN models (similar Pearson on training/testing data), while the RF + N model looks to be partially over-fitted (although with best test metrics). Source data are provided in the Source Data file.

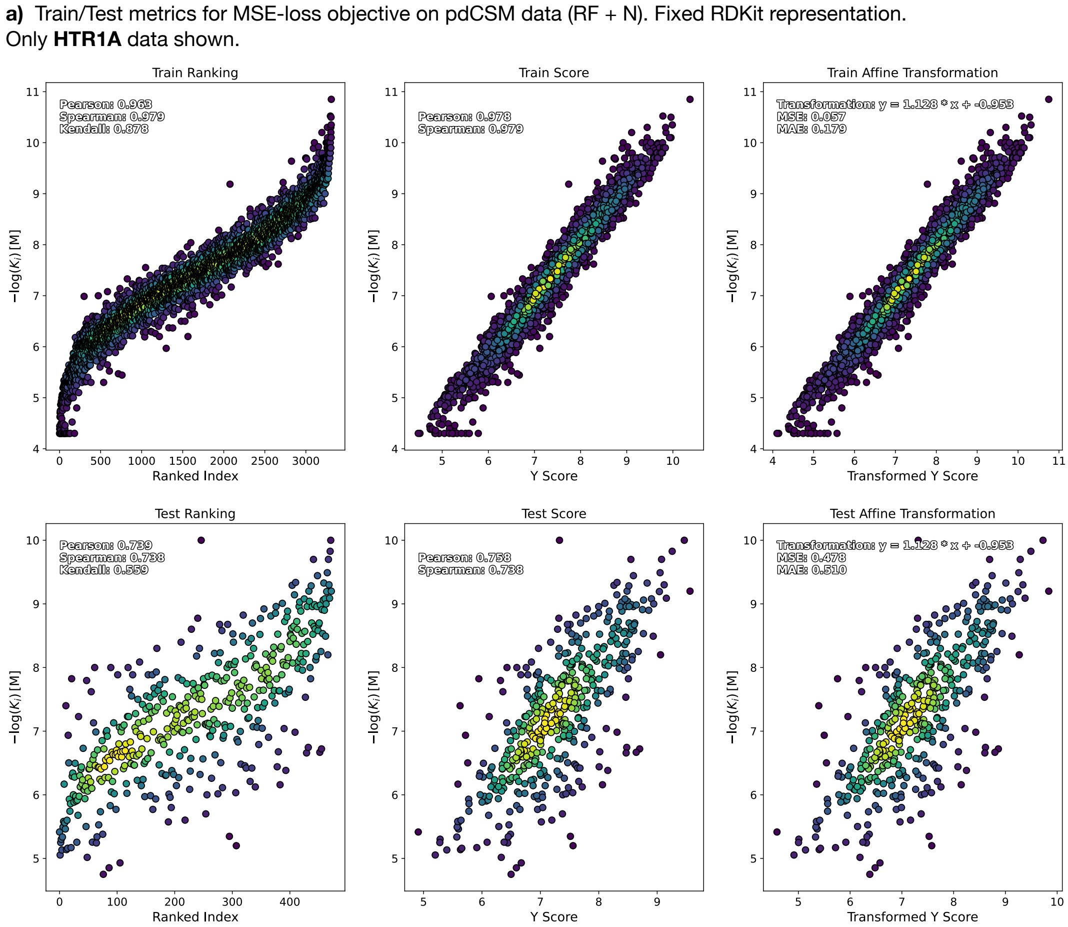

Figure S15: Train/Test metrics for the pdCSM dataset using the RF + N model, a MSE-loss, and fixed RDkit compound representations (copying those from the original paper, without feature selection). The model is trained on all data and only training and testing data points for DTIs involving the HTR1A hGPCR is shown. Source data are provided in the Source Data file.

a) NN_θ^6 test metrics for different loss-objectives and model types.

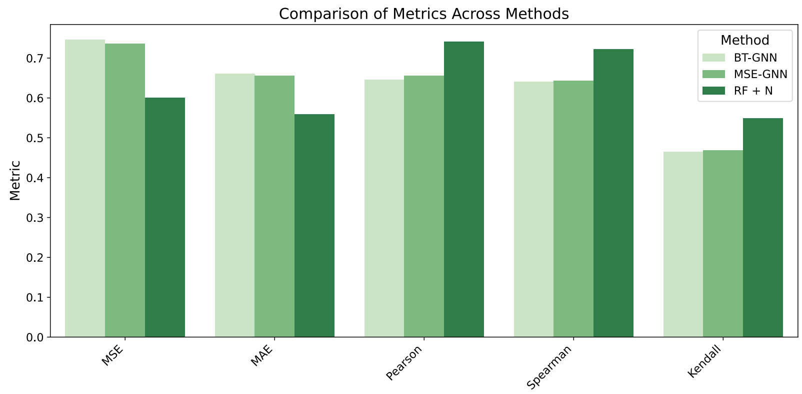

b) Learned (transfer learning) representation vs. fixed RDkit representation test Pearson correlation show slightly increase performance of learned molecule representation.

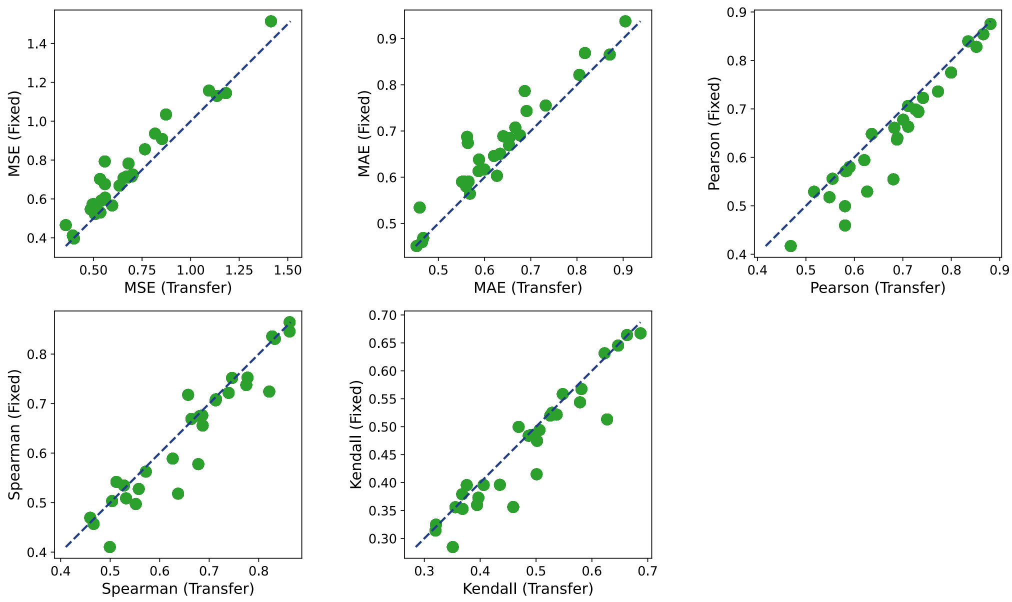

Figure S16: **(a)** A comparison of test metrics between ML models on the pdCSM dataset show that the RF + N model outperforms the others. **(b)** We compare the node-embedding (compound representation) for the same model (size and training objective, training epochs), which shows that learned representations (obtained from ChemProp) slightly outperform the fixed RDKit representation. Source data are provided in the Source Data file.

a) Train/Test metrics for RF + N on pdCSM data. Fixed RDKit representation. Only **CHRM3** data shown.

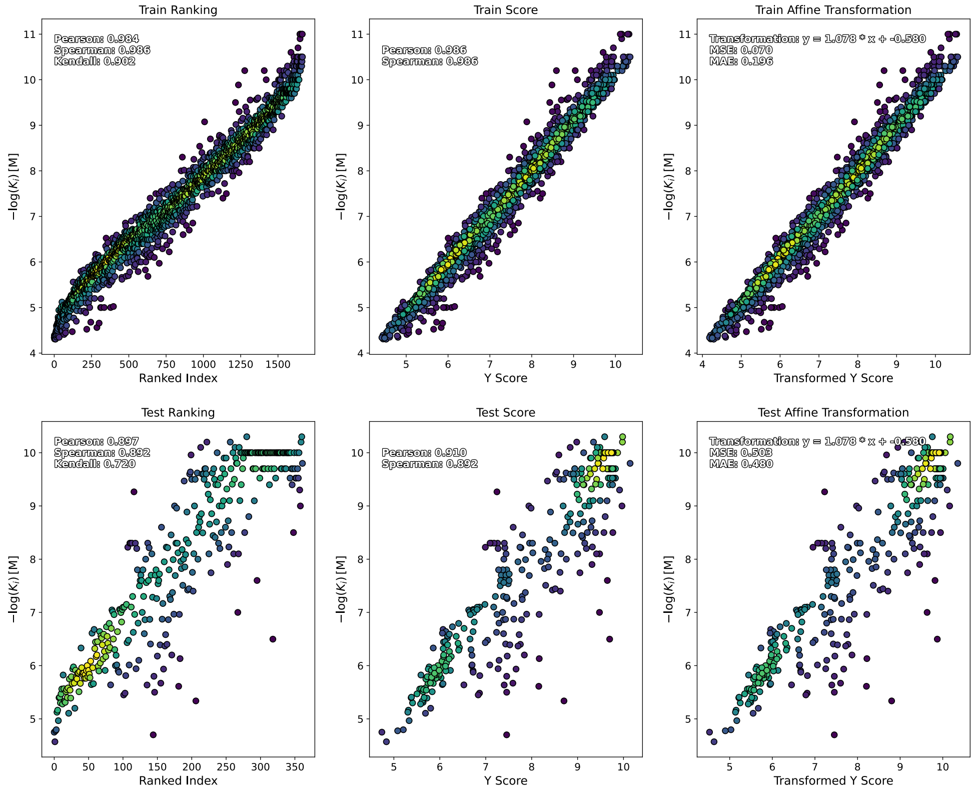

b) Train/Test metrics for MSE-loss objective on pdCSM data (CSNN). Fixed RDKit representation. Trained only on **CHRM3** data shown (rather than all data with receptor representation, above). High variance across epochs, lower test metrics.

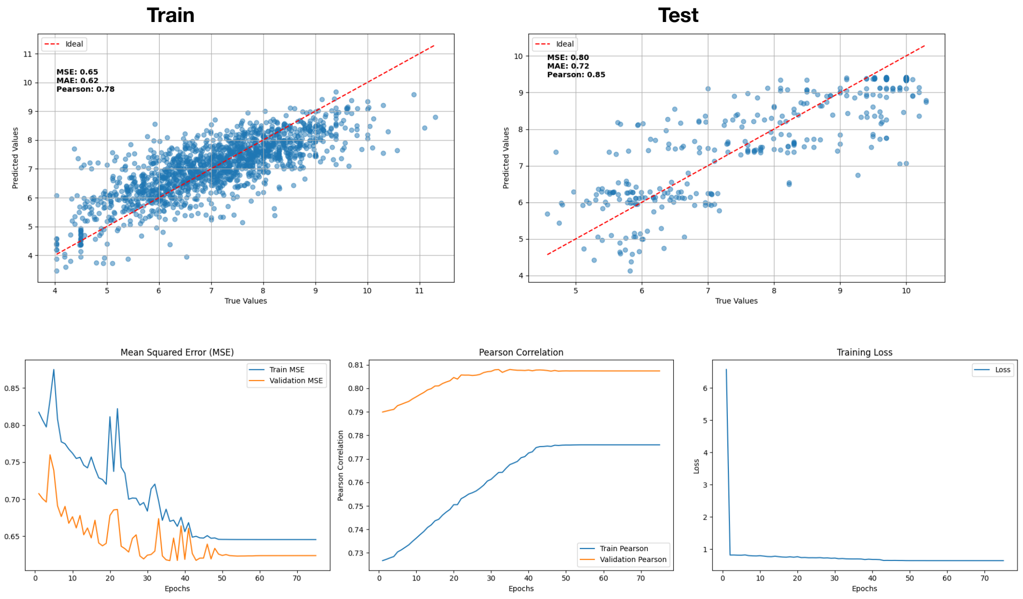

Figure S17: **Comparing one-target-one-model vs One-model-for-all**. The original pdCSM method (*26*) trains a RF model per hGPCR (thus avoiding an explicit target representation). We compare the settings (a) and (b) and found that training was unstable due to a high-variance (small batches and little data). A single model which handles all data, necessarily representing both the compound and target representations was found to be the most efficient solution. Source data are provided in the Source Data file.

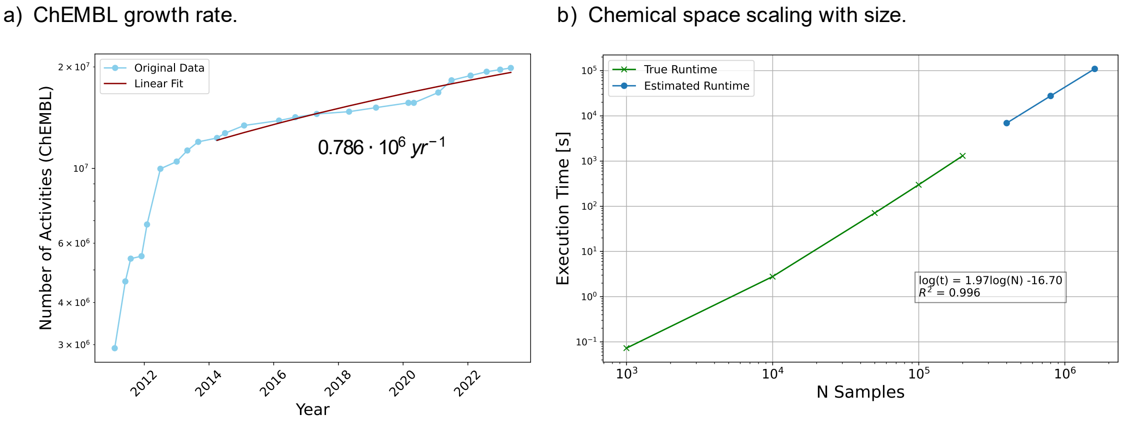

Figure S18: **(a)** ChEMBL growth rate per year estimated using data from (*27*), which documents activites for each ChEMBL release. **(b)** Chemical space network construction computation time, as calculated on a home computer (MacBook, Apple M1 Max, 32 GB RAM). For tens of thousands of compounds, the execution time is on the order of seconds; for hundreds of thousands, it is on the order of minutes. For compound libraries larger than 1 million compounds the runtime was estimated, but could not be executed due to lacking memory. Although, looking up the 1-hop neighbourhood for a single compound is extremely fast for a 1.6 million compound library 1.55 ± 0.05 seconds (using randomly samples ZINC smiles strings with precomputed fingerprints). For less than 20K compounds, a batch all-vs-all CSN can be computed in less than 3.9 seconds. For larger libraries a one-vs-all calculation is performed for all rows (upper-triangular). Source data are provided in the Source Data file.

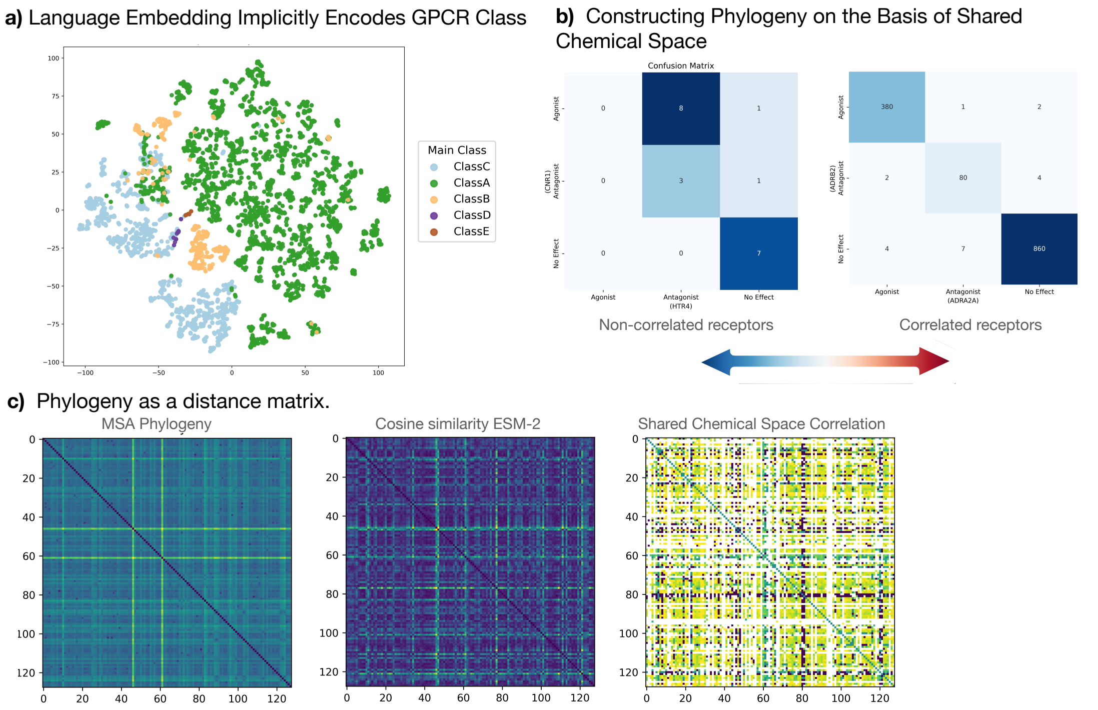

Figure S19: **(a)** Protein Language model (ESM-2) embedding of hGPCR using the ground truth labels from the GDS dataset (*28*), which is comprised of 8354 unique GPCRs. Embedding were dimensionality reduced using t-SNE (*29*). **(b)** Our unique labeled dataset allows for the identification of correlated hGPCRs on the basis of a shared chemical space. For example, ADRA2A and ADRB2 are known to be related phylogenetically by MSA, which is reflected in the populated diagonal, implying that a compound that is an agonist on one hGPCR is likely also to be on the other. For less correlated hGPCRs, the chemical space will be less shared (off-diagonal entries), from which a metric can be derived using Cramer's V to obtain a correlation between categorical variables. **(c)** The output of all three methods (i) MSA, (ii) ESM-2 embeddings, and (iii) shared chemical space are a distance matrix from which the phylogenetic trees in S20 are derived. Source data are provided in the Source Data file.

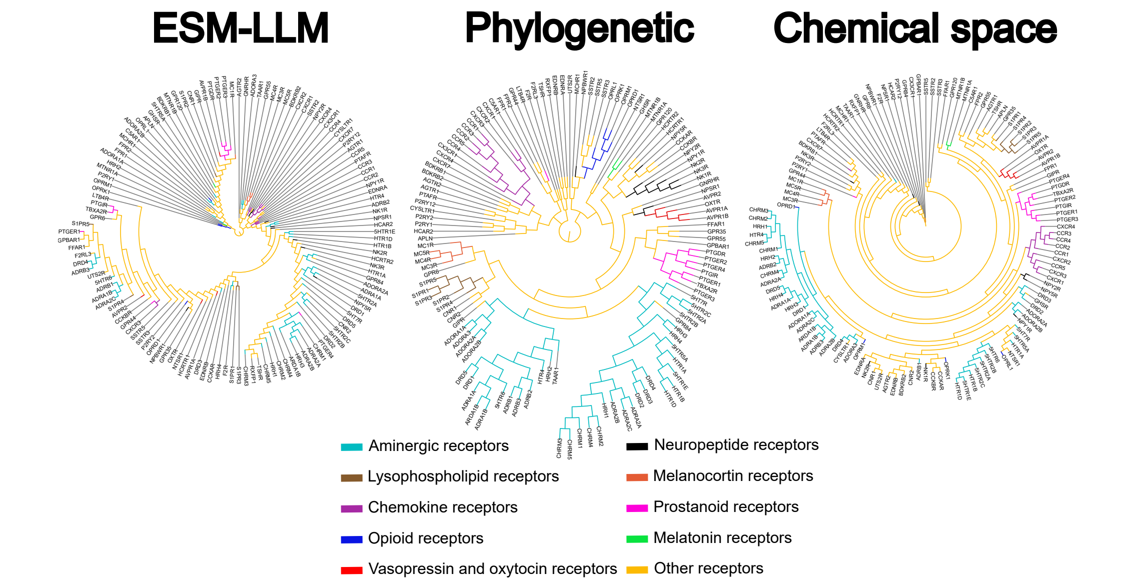

Figure S20: hGPCR clustering showing tree structures generated from distances calculated by the ESM-2 Large Language model (ESM-LLM), multiple sequence alignment (MUSCLE), and from the chemicals they are able/unable to sense. Phylogenetic trees are based on distance matrices (See Figure S19)

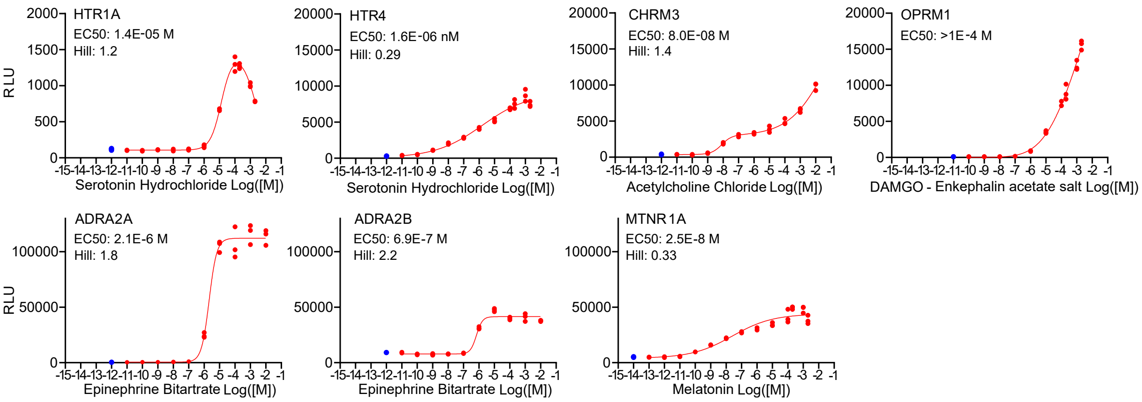

Figure S21: Dose responses of 7 chosen hGPCRs with known agonists. Red dots are individual replicates at each concentration (n=3 biological replicates), blue dots are 0 M negative controls (n=3 biological replicates), not included in the generation of the DRCs. All DRCs are generated using built-in functions in GraphPad Prism 9.5.0. For OPRM1 The EC50 is given as a lower bound, 50% of the maximum observed RLU of this hGPCR as we were unable to plateau reporter output at high concentrations. hGPCR independent effects were determined using the parent strains (ScFH236 & 237)(Supplementary Figure S22). Source data are provided in the Source Data file.

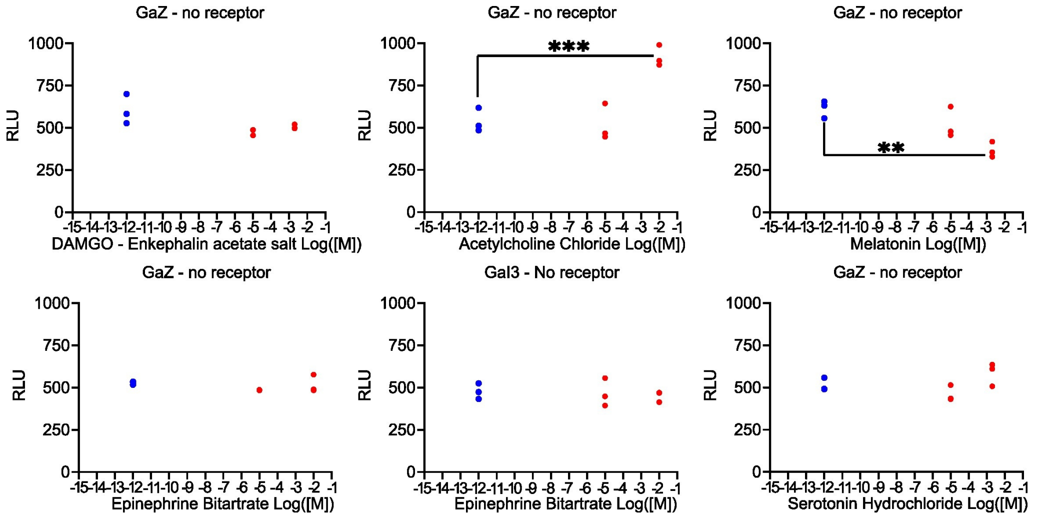

Figure S22: **No-hGPCR controls for Figure 2**, Blue dots are 0-concentration controls placed artificially on the logarithmic scaled x-axis. Red dots represent individual datapoints (N=3 biological replicates at all tested concentrations) of no-hGPCR control strains tested against concentrations of the ligands used in Figure 4d and S21. hGPCR independent effects are observed for acethylcholine chloride at 10 mM and for melatonin at 2 mM (Supplementary figure S22). The early response of acetylcholine chloride against CHRM3 (1E-10 – 1E-5 M) plateaus before the unspecific effect begins, allowing us to determine an EC50 and Hill coefficient for the specific effect on the hGPCR. For MTNR1A, the receptor unspecific effect of melatonin indicates that MTNR1A mediated signalling might not have reached a true plateau. *p=0,012, ***p=0.0002 two-tailed paired students t-test. Source data are provided as a Source Data file.

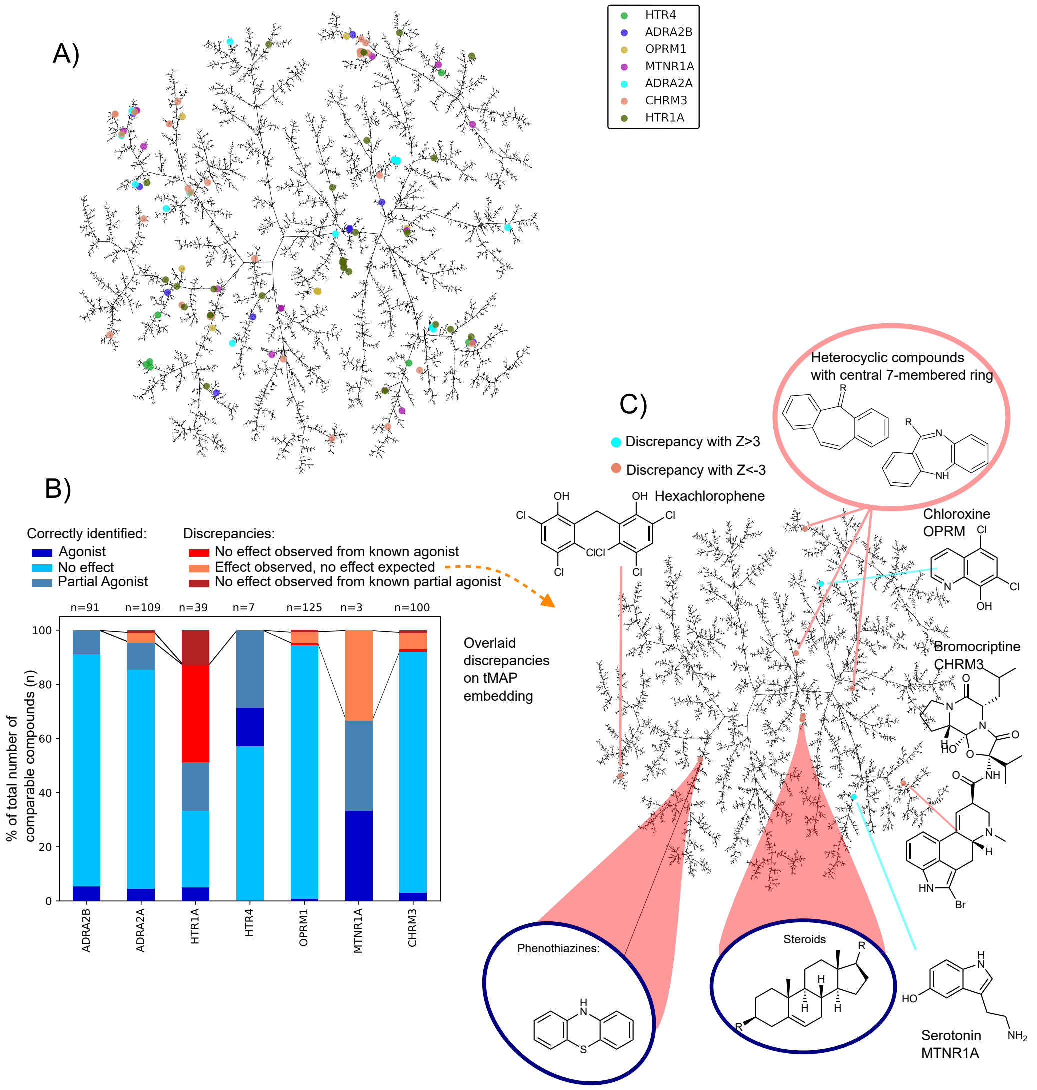

Figure S23: tMAP embedding (*30*), generated on the dataset of chemicals targeting one or more of the 128 investigated GPCRs. A) The hits obtained from the tested chemical library is highlighted colored by which GPCR the hit was observed on. B) hit discrepancies identified by 1:1 comparison with prior art. Source data are provided as a Source Data file. C) Discrepancies, where no effect was expected, but an effect was observed, was overlaid on the tMAP of the chemical space. Some discrepancies clustered together. Source data are provided in the Source Data file.

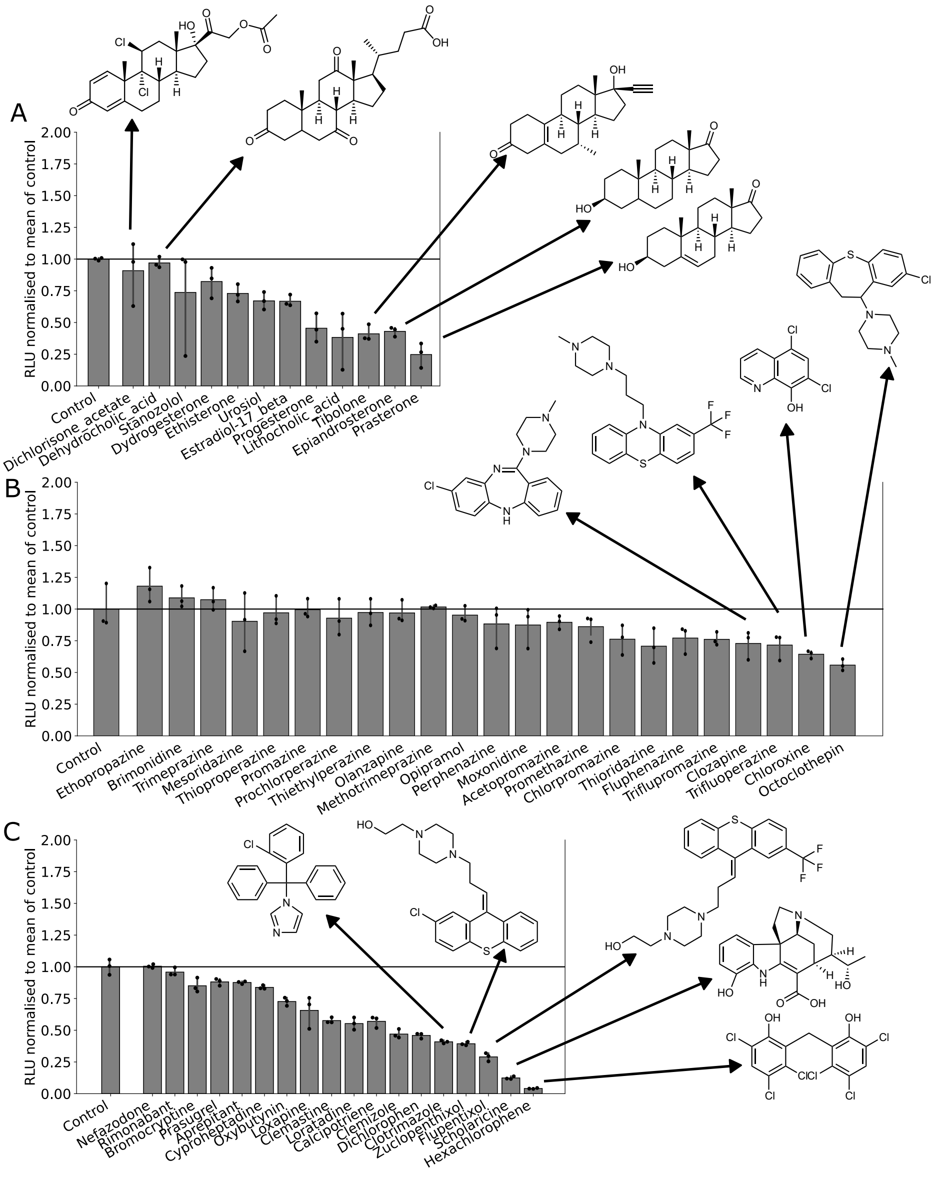

Figure S24: GPCR independent effects on yeast, assessed as RLU normalised to the mean RLU of negative controls (1% DMSO) n=3. **A)** measurements of steroid class compounds, showing that more oxidised steroids like Dichlorisone and dehydrocholic acid do not affect the signal dampening in the same way as more reduced steroids. **B)** Measurements for phenothiazines and similar compounds, illustrating that especially halogenated phenothiazines and 7-membered ring containing compounds dampen the signal. Chloroxine was also included here, as it dampened the signal on non-OPRM1 receptors. **C)** The bottom panel contains more tri-cyclic compounds aswell as other compounds selected due to observations of generally dampening effects on luminescence. Compound structures rendered for the 5 most inhibiting compounds in each plot, and for other compounds of interest. Any compound where the standard deviation does not reach the mean of the controls is removed from further analysis due to bias from receptor independent effects. Controls in each plot represent separate triplicates of measurements on RLU upon exposure to 1% DMSO, which is present in all samples. N=3 for all bars. Source data are provided in the Source Data file.

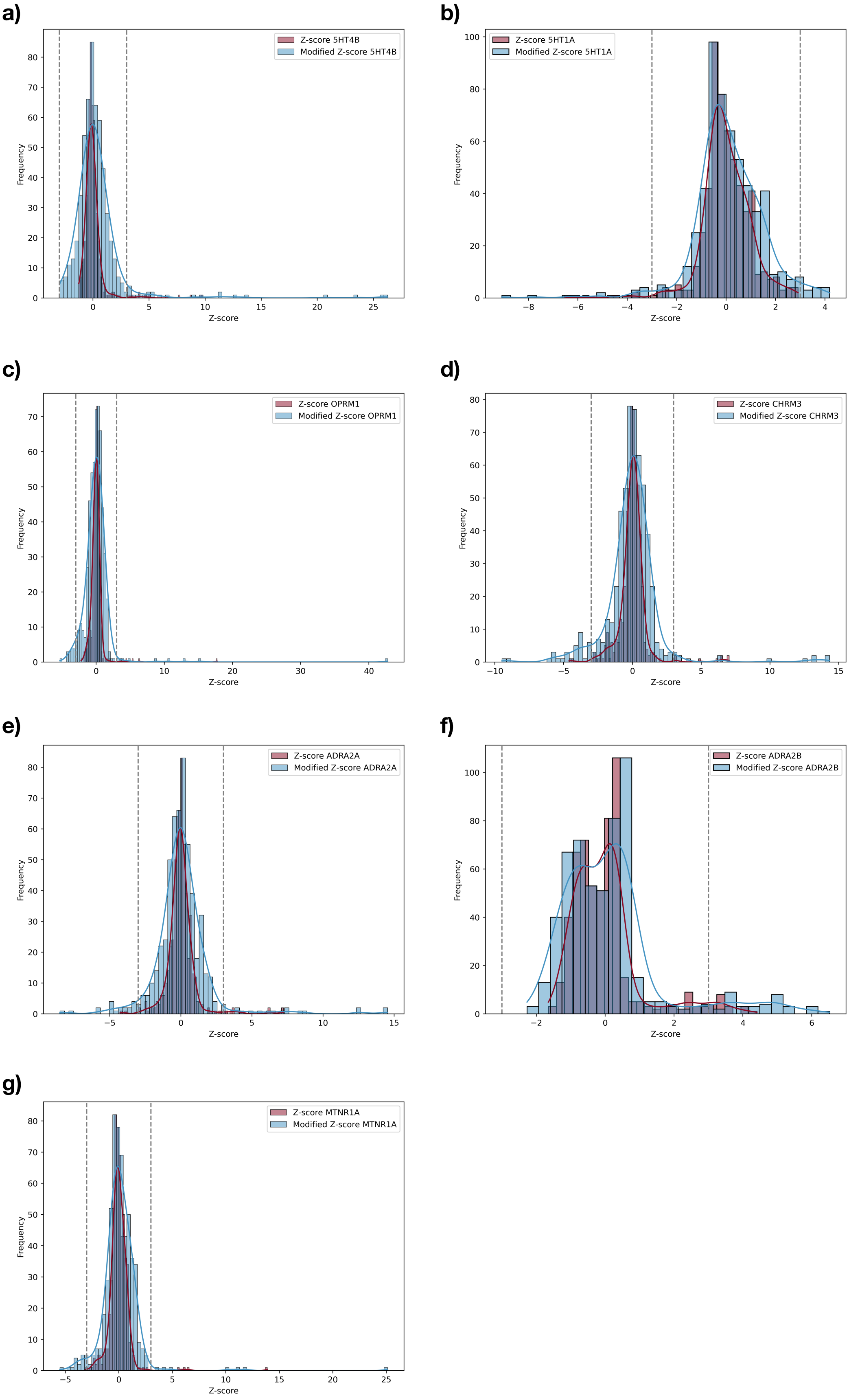

Figure S25: **Effect of Modified Z-score on recovering hits**, data is shown for each hGPCR and the histogram fit is plotted using kernel density estimation to contrast the effect of the Z-score (Eq. (9)) against the modified Z-score (Eq. (10)). Panels **a), b), c), d), e), f), and g)** corresponds to receptors 5HT4b, 5HT1A, OPRM1, CHRM3, ADRA2A, ADRA2B, and MTNR1A respectively. Source data are provided in the Source Data file.

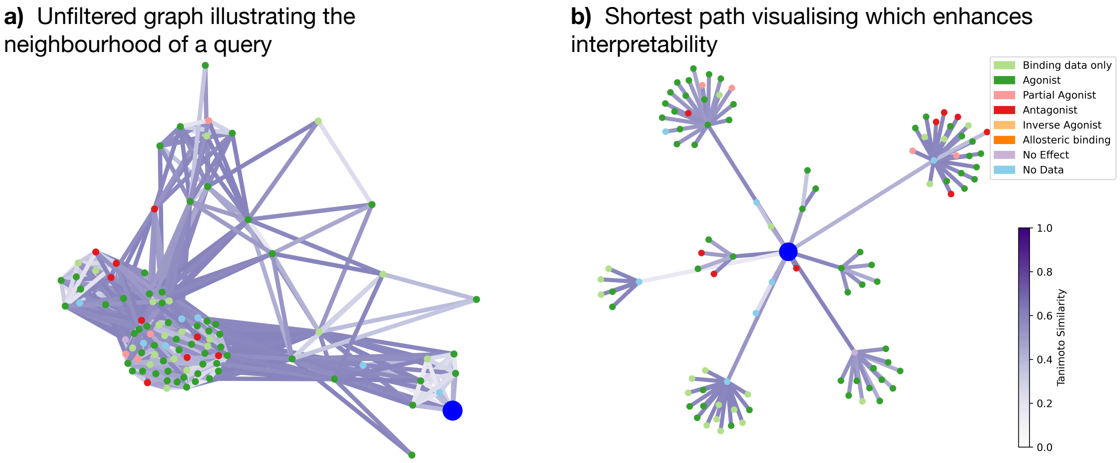

Figure S26: Enhancing interpretability of graph neighbourhoods for a given query by considering only the shortest paths from the query to all other codes. This converts the graph into a tree graph that all expand radially from the query. **a)** Shows the unfiltered graph, while **b) visualizes the benefit to interpretation from only considering the shortest path**

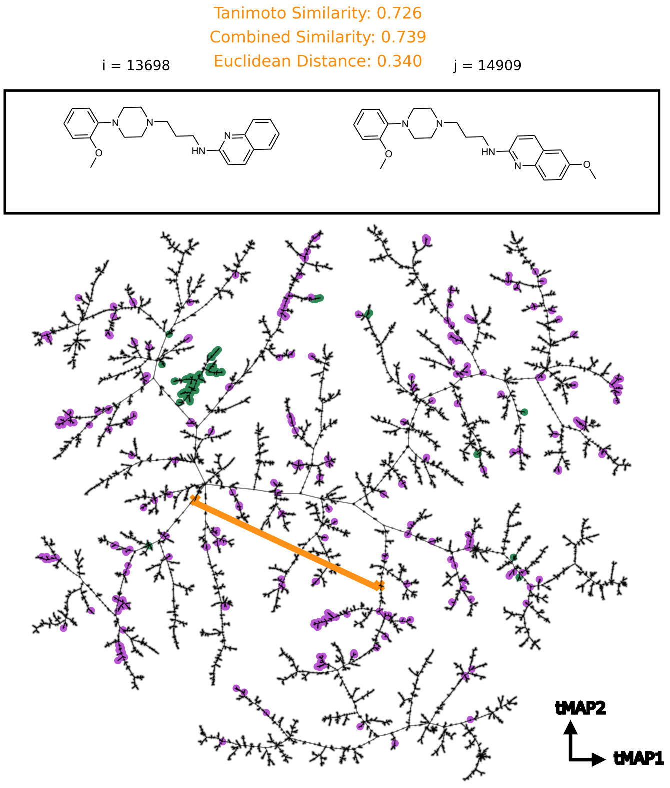

Figure S27: To avoid a graph structured chemical space an embedding space can be used to efficiently find k-nearest neighbours. We tried using the tMAP (*30*) projection, which we use for visualising chemical space, and which is developed for this chemical use case, but found that molecules which have a high Tanimoto similarity do not necessarily lie close in the embedding space. This makes finding k-NN problematic and as such a all-vs-all pairs similarity search was carried out.

S10 Supplementary Tables

Table S1: Performance metrics under the homophilly assumption for pdCSM dataset regression task: the mean value over the chemical neighbourhood is a good approximation of the true value. Sorted in order of descending Pearson correlation coefficient.

Method	hGPCR	MSE	MAE	Pearson	Kendall	Spearman
mean	CHRM3	0.694	0.634	0.87	0.664	0.862
mean	NK2R	0.507	0.557	0.863	0.63	0.815
mean	CCR3	0.397	0.464	0.839	0.621	0.828
mean	S1PR5	0.434	0.383	0.837	0.775	0.896
mean	EDNRB	0.455	0.546	0.815	0.593	0.788
mean	OPRM1	0.835	0.69	0.765	0.569	0.762
mean	ADRA1A	0.648	0.639	0.745	0.553	0.74
mean	HRH3	0.579	0.587	0.72	0.489	0.673
mean	5HTR6	0.729	0.651	0.716	0.527	0.727
mean	HTR1A	0.537	0.57	0.713	0.514	0.698
mean	BDKRB1	1.22	0.816	0.701	0.361	0.511
mean	P2RY1	0.535	0.457	0.693	0.471	0.644
mean	ADORA1A	0.665	0.59	0.688	0.509	0.687
mean	ADORA2B	0.571	0.574	0.68	0.507	0.686
mean	ADORA3	0.824	0.687	0.678	0.494	0.674
mean	5HTR2C	0.577	0.58	0.668	0.487	0.664
mean	GPR35	1.306	0.856	0.663	0.619	0.786
mean	GNRHR	0.882	0.695	0.608	0.441	0.621
mean	GPBAR1	1.125	0.862	0.607	0.462	0.651
mean	CHRM4	0.573	0.583	0.598	0.453	0.64
mean	DRD4	0.56	0.577	0.592	0.421	0.578
mean	SSTR5	0.849	0.726	0.585	0.398	0.562
mean	PTGER1	0.741	0.689	0.567	0.386	0.516
mean	CHRM5	0.392	0.462	0.561	0.332	0.479
mean	MCHR1	0.543	0.56	0.554	0.378	0.527
mean	GPR44	0.742	0.67	0.554	0.376	0.535
mean	HCAR2	0.669	0.626	0.532	0.321	0.456
mean	S1PR3	0.58	0.622	0.508	0.345	0.487
mean	MTNR1A	1.41	0.918	0.419	0.269	0.393

Table S2: Tasks performed by state of the art predictive algorithms for DTI predictions

Input	Task	Explanation	Notation	Type	Refs
ci	*yi*	Does the compound have GPCR activity?	$P(yi \mid ci)$	Regression, Classification	(*31*, *32*))
ci,tj	*yi*	Given a compound and its target representation, is the compound active?	$P(yi \mid ci,tj)$	Regression, Classification	
ci,cj	*yi,yj*	Given two compounds, how active are they?	$P(yi,yj \mid ci,cj)$	Regression, Classification	(*33*)
ci	*tj*	Given a compound, predict its target.	$P(ti \mid ci)$	Classification	(*34*)
ci					(*35*)
Inference from Neighbourhood	*aj*	Given a compound, predict adverse drug events.	$P(ai \mid ci)$	Regression, Classification	
ci,tj,Ni(yj)	*yi*	Given a compound, it's neighbourhood activity on one target j, and a target representation tj, is it active?	$P(yi \mid ci,Ni(yj),tj)$	Classification	(ours)
ci,Ni(yn),tn	*yi,n*	Given a compound, it's neighbourhood activity on all n targets, and target representations, on which target is ci active?	$P(yn,i \mid ci,Ni(yn),tj)$	Classification	(ours)

**Class**	**Precision**	**Recall**	**F1-Score**	**Support**
Agonist	0.9256	0.8746	0.8994	61835
No Effect	0.9120	0.9312	0.9215	57575
Antagonist	0.8940	0.9338	0.9135	51418
Inverse Agonist	0.7745	0.7058	0.7386	1499
Allosteric Binding	0.7046	0.6621	0.6827	4208
Partial Agonist	0.8179	0.9119	0.8623	2621
Accuracy			0.9039	179156
Macro Avg	0.8381	0.8365	0.8363	179156
Weighted Avg	0.9042	0.9039	0.9036	179156

Table S3: Argmax(.) performance metrics across all 128 hGPCR. Metrics are shown per bioactivity class, and the data illustrates the remarkable predictability of a compound given its neighborhood.

Table S4: The 7 hGPCRs investigated in vitro in this study, and the known treatable conditions for which the respective targets have been used clinically.

hGPCR Target	Known target indications
HTR1A	Anxiety(*36*)<br>Depression(*36*)<br>Pain(*36*)<br>Schizophrenia(*36*)<br>Neuroprotection(*36*)
HTR4	Gastro-intestinal disorders(*37*)<br>Anxiety(*38*)
CHRM3	Mucous membrane dryness (*39*)<br>Blood pressure regulation(*40*)<br>Alzheimers disease(*41*)<br>Schizophrenia(*41*)
OPRM1	Pain(*42*)<br>Addiction(*42*)
MTNR1A	Sleep–Wake Disorders(*43*)
ADRA2A & ADRA2B	Pain(*44*)<br>Peri-operative ischaemia(*44*)<br>Blood pressure regulation(*44*)

Table S5: **a.** Prices estimated for the costs of performing an assay similar to the one described in this paper in commercially available GPCR screening systems. **b.** We used a low volume 96wp format for our small screen of 4000 compounds, converting to 384 wp format as demonstrated by Miettinen et al 2022.(*54*) will reduce the cost of plates by up to 55%, lowering the total cost pr. well to 0.91USD. **c.** Not all suppliers state their prices outside confidential quotes, this list only contains suppliers with listed prices. Other suppliers include Multispan, Molecular Devices, and Innoprot. **d.** For the costs of multiplexing, the prices estimated in the respective publications were used, these values reflect the price pr. well, where a single well can assay more than 300 GPCRs. The supporting calculations are available in supplementary file S1. The prices listed are for the operational costs only, excluding instrument acquisition, overhead, and salary. Other sources estimate costs around 10 times higher for mammalian cell based assays(*53*)

Assay	Advantages	Limitations	Costs: 4000 tests across 7 receptors(USD/well) (**a**) (**c**)
cAMP	Simple to perform.<br>High sensitivity<br>Non-lytic methods available for real-time measurements(*45*)	Gi/o or Gs coupled receptors or chimeric g-proteins only.	PerkinElmer:<br>Alphascreen - 1.79$<br>LANCE - 3.21$<br>Cisbio:<br>HTRF® - 1.97$<br>Eurofins:<br>HitHunter™ - 6.73$
IP1	More robust alternative to Ca2+ mobilisation assays(*46*)	Gq or Gi coupled receptors or chimeric g-proteins only	Cisbio:<br>HTRF-IP-One® - 2.84$
Ca2+ mobilization	Cheap compared to most other mammalian cell line based assays.	Rapid and transient signal not suitable for slow binding.<br>Gq or Gi coupled receptors or chimeric g-proteins only(*47*)	Eurofins:<br>ChemiBrite™- 1.80$
β-arrestin recruitment	Can be used to study biased signalling<br>G-protein independent - very applicable to deorphanization<br>High sensitivity, broad dynamic range.(*48*)	Does not determine Ga-mediated signaling.<br>Most versions of this assay require receptor editing.(*48*)	Eurofins:<br>PathHunter®-11.7$<br>ThermoFischer:<br>Tango™ - 8.58$
Receptor internalisation	Studies biased signaling<br>G-protein independent - very applicable to deorphanization(*49*)	Does not determine Ga-mediated signaling.	Eurofins:<br>PathHunter® -11.4$
GTPγ[35S] /Eu binding.	Works with any Ga coupling.<br>Fast and simple protocol.(*50*)	Sensitivity lower in HTS format.<br>May require radioactive substances(*50*)	PerkinElmer - 5.29$
Radioligand binding	High sensitivity, broad dynamic range.<br>Fast and simple protocol(*51*, *52*).	GPCR must have a known ligand.<br>May require radioactive substances<br>Can not determine receptor signalling.	PerkinElmer:<br>Radioligand competition - 5.89$<br>Cisbio:<br>HTRF Tag-lite - 2.96$
Multiplexed screening	Very high throughput<br>low ligand requirements	Requires biased signalling in mammalian cell assays(*53*).<br>Requires Ga-signalling in yeast cell assays(*22*).	PRESTO-Salsa-17$(**d**)(*53*)
Yeast biosensor	High dynamic range.<br>Fast and simple protocol.<br>Significantly cheaper than mammalian cell lines	GPCR must be functionally signalling in yeast.	Our work -1.01$ **b**

Table S6: **overview of EC50, Hill coefficient and detection limits observed compared to previously reported assays.** Lowest EC50's reported for each hGPCR for the same ligand as was used in this study. **a.** The only reported data in ChEMBL on ADRA2B against epinephrine is from a radioligand competition assay, and as such the value reported is a K_i corresponding to competition with [3H]Rauwolscine.

hGPCR	EC50 [M] (This study)	Detection limit [M] (This study)	Hill Coefficient (This study)	Lowest EC50 [M] (Reported in ChEMBL)	Assay type (Reported in ChEMBL)
ADRA2A	2.1E-06	1.0E-08	1.80	3.0E-08 (*55*)	[35S]GTPγS
ADRA2B	6.9E-07	1.0E-07	2.20	4.0E-7 (*56*)	Radioligand competition[a]
HTR4	1.6E-06	1.0E-10	0.29	1.2E-08 (*57*)	cAMP
HTR1A	1.4E-05	1.0E-06	1.20	5.0E-10 (*58*)	[35S]GTPγS
CHRM3	8.0E-08	1.0E-09	1.40	5.6E-08 (*59*)	cAMP
MTNR1A	2.5E-08	1.0E-11	0.33	2.2E-11 (*60*)	cAMP
OPRM1	>1.0E-05	1.0E-07	ND	3.0E-10 (*61*)	cAMP

Table S7: Genes used in this study

Gene name	accesion	Codon opti-mised?	Sequence
HTR4	Q13639-1	Yes	ATGGATAAATTGGATGCTAATGTTTCTTCTGAAGAAGGTTTTGGTTCTGTTGAAAAAG<br>TTGTTTTGTTGACTTTTTTGTCTACTGTTATTTTGATGGCTATTTTGGGTAATTTGTTGG<br>TTATGGTTGCTGTTTGTTGGGATAGACAATTGAGAAAAATTAAAACTAATTATTTTAT<br>TGTTTCTTTGGCTTTTGCTGATTTGTTGGTTTCTGTTTTGGTTATGCCATTTGGTGCTAT<br>TGAATTGGTTCAAGATATTTGGATTTATGGTGAAGTTTTTTGTTTGGTTAGAACTTCTT<br>TGGATGTTTTGTTGACTACTGCTTCTATTTTTCATTTGTGTTGTATTTCTTTGGATAGAT<br>ATTATGCTATTTGTTGTCAACCATTGGTTTATAGAAATAAAATGACTCCATTGAGAAT<br>TGCTTTGATGTTGGGTGGTTGTTGGGTTATTCCAACTTTTATTTCTTTTTTGCCAATTA<br>TGCAAGGTTGGAATAATATTGGTATTATTGATTTGATTGAAAAAAGAAAATTTAATC<br>AAAATTCTAATTCTACTTATTGTGTTTTTATGGTTAATAAACCATATGCTATTACTTGT<br>TCTGTTGTTGCTTTTTATATTCCATTTTTGTTGATGGTTTTGGCTTATTATAGAATTTAT<br>GTTACTGCTAAAGAACATGCTCATCAAATTCAAATGTTGCAAAGAGCTGGTGCTTCTT<br>CTGAATCTAGACCACAATCTGCTGATCAACATTCTACTCATAGAATGAGAACTGAAA<br>CTAAAGCTGCTAAAACTTTGTGTATTATTATGGGTTGTTTTTGTTTGTGTTGGGCTCCA<br>TTTTTTGTTACTAATATTGTTGATCCATTTATTGATTATACTGTTCCAGGTCAAGTTTG<br>GACTGCTTTTTTGTGGTTGGGTTATATTAATTCTGGTTTGAATCCATTTTTGTATGCTT<br>TTTTGAATAAATCTTTTAGAAGAGCTTTTTTGATTATTTTGTGTTGTGATGATGAAAG<br>ATATAGAAGACCATCTATTTTGGGTCAAACTGTTCCATGTTCTACTACTACTATTAAT<br>GGTTCTACTCATGTTTTGAGAGATGCTGTTGAATGTGGTGGTCAATGGGAATCTCAAT<br>GTCATCCACCAGCTACTTCTCCATTGGTTGCTGCTCAACCATCTGATACTTGA
HTR1A	P08908	Yes	ATGGACGTATTATCACCTGGACAGGGCAACAACACAACAAGTCCCCCTGCACCTTTC<br>GAGACAGGCGGGAACACAACAGGCATCAGTGACGTGACAGTATCATACCAGGTGAT<br>CACATCCTTACTACTTGGGACATTAATATTCTGCGCCGTCCTGGGAAACGCATGCGTA<br>GTGGCAGCCATAGCCCTGGAGAGGAGTCTGCAGAACGTCGCAAACTACCTGATCGGC<br>AGCTTAGCCGTAACAGACTTAATGGTCTCAGTGTTAGTGCTTCCGATGGCCGCATTAT<br>ACCAGGTGCTAAACAAGTGGACGCTGGGACAGGTGACATGCGACCTTTTCATCGCGT<br>TAGACGTCTTATGCTGCACATCATCGATCCTTCACCTTTGCGCAATCGCCCTTGACCG<br>TTACTGGGCCATCACCGACCCCATCGACTACGTGAACAAGAGGACCCCTAGGCGTGC<br>GGCCGCATTAATATCGCTGACCTGGTTAATCGGATTCCTAATATCCATACCGCCTATG<br>TTAGGATGGAGAACCCCGGAAGACCGTAGCGACCCCGACGCCTGCACGATAAGCAA<br>GGACCACGGATACACCATCTACAGTACATTCGGAGCATTCTACATCCCTTTATTACTA<br>ATGTTAGTACTTTACGGCCGTATATTCCGTGCCGCAAGGTTCAGGATCCGTAAGACA<br>GTAAAGAAGGTCGAGAAGACCGGAGCAGACACAAGACACGGGGCCAGTCCTGCTCC<br>GCAGCCAAAGAAGAGTGTGAACGGCGAGAGTGGGTCGAGGAATTGGAGACTGGGCG<br>TCGAGTCGAAGGCCGGCGGAGCATTATGTGCTAACGGGGCGGTCAGGCAAGGTGAC<br>GACGGCGCAGCGTTAGAGGTAATAGAGGTCCACAGGGTCGGAAACAGCAAGGAGCA<br>CTTACCTTTACCTTCTGAGGCCGGACCTACCCCGTGCGCGCCTGCCAGCTTCGAGAGG<br>AAGAACGAGAGGAACGCAGAGGCGAAGCGTAAGATGGCCTTAGCAAGGGAGCGTA<br>AGACAGTGAAGACACTAGGCATCATAATGGGAACCTTCATACTATGCTGGTTACCTT<br>TCTTCATAGTCGCCCTTGTCCTTCCCTTCTGCGAGTCGTCCTGCCACATGCCGACATTA<br>CTTGGGGCAATCATCAACTGGCTTGGCTACAGTAACTCCCTTCTAAACCCCGTGATCT<br>ACGCCTACTTCAACAAGGACTTCCAGAACGCCTTCAAGAAGATAATCAAGTGCAAGT<br>TCTGCCGTCAGTAG

Continued on next page

Table S7: Genes used in this study (Continued)

ADRA2A	P08913	Yes	ATGTTCCGGCAGGAGCAGCCACTCGCCGAGGGTTCCTTTGCCCCCATGGGAAGTCTT<br>CAGCCTGACGCCGGCAACGCTAGCTGGAATGGCACCGAAGCGCCCGGTGGAGGGGC<br>TCGGGCTACCCCATATTCTCTCCAGGTCACACTGACCCTGGTTTGTCTGGCCGGTCTC<br>CTGATGCTGCTGACGGTCTTTGGCAATGTTTTGGTGATAATTGCGGTGTTCACCTCCA<br>GAGCACTCAAGGCTCCCCAGAACCTGTTCTTGGTCAGCCTGGCGTCTGCCGATATTCT<br>GGTCGCCACCCTCGTAATTCCGTTTTCTCTTGCCAATGAGGTTATGGGTTACTGGTAT<br>TTTGGTAAGGCATGGTGCGAGATATACCTTGCTCTCGACGTTCTCTTCTGCACCAGCA<br>GTATTGTGCATCTGTGCGCAATTAGTCTGGACAGATATTGGTCCATCACGCAGGCAA<br>TAGAATATAACTTGAAAAGGACTCCTCGGCGCATTAAGGCTATCATCATCACTGTCT<br>GGGTCATTAGCGCTGTGATAAGCTTCCCTCCACTTATTTCCATTGAAAAAAAAGGAG<br>GAGGCGGAGGGCCGCAGCCTGCGGAGCCCCGCTGTGAGATCAACGATCAGAAATGG<br>TATGTCATCTCCTCCTGCATTGGGTCCTTCTTCGCCCCATGTCTGATTATGATCCTCGT<br>ATATGTTAGGATTTATCAGATAGCTAAGCGAAGGACCCGGGTCCCCCCATCCAGGAG<br>AGGTCCAGACGCAGTGGCCGCTCCCCCCGGAGGCACAGAAAGGAGACCTAACGGTC<br>TGGGACCAGAGCGCTCCGCAGGACCTGGAGGTGCAGAAGCCGAGCCCTTGCCGACA<br>CAACTGAACGGAGCTCCCGGCGAACCAGCCCCAGCAGGTCCTCGCGATACTGACGCA<br>CTGGACCTCGAAGAGTCCTCTAGCAGCGATCATGCAGAGCGCCCTCCAGGGCCTAGG<br>AGACCTGAAAGAGGACCCAGAGGTAAAGGCAAGGCCCGGGCAAGTCAAGTGAAACC<br>GGGAGACTCTCTGCCTCGGAGGGGTCCAGGTGCTACTGGTATAGGGACTCCTGCCGC<br>CGGTCCCGGAGAAGAGCGGGTCGGCGCCGCTAAGGCAAGTCGCTGGCGGGGCAGAC<br>AAAACCGGGAAAAAAGGTTTACATTCGTGCTCGCGGTAGTGATTGGCGTCTTCGTCG<br>TGTGCTGGTTTCCATTCTTTTTACCTATACCCTGACAGCCGTCGGCTGTTCTGTTCCA<br>CGGACACTGTTCAAGTTCTTTTTCTGGTTTGGCTACTGTAACTCAAGCCTGAACCCGG<br>TAATCTATACAATATTTAACCACGATTTTCGGAGGGCTTTTAAAAAGATCCTGTGCAG<br>AGGCGATAGGAAACGAATCGTTTAA
ADRA2B	P18089	Yes	ATGGATCATCAGGATCCCTATTCTGTCCAGGCCACTGCGGCCATCGCTGCCGCTATCA<br>CTTTTCTCATTCTCTTTACCATCTTCGGGAACGCGCTTGTAATTCTGGCGGTGCTCACC<br>AGCAGGTCACTCAGGGCACCACAAAACCTTTTTCTGGTCTCCCTCGCGGCAGCTGAC<br>ATTCTCGTCGCAACCCTCATCATTCCCTTTTCACTCGCTAATGAGTTGTTGGGATATTG<br>GTATTTTCGCCGAACCTGGTGCGAGGTGTATCTCGCCCTGGACGTACTGTTTTGCACC<br>TCTTCAATTGTCCATCTTTGTGCAATCAGCCTGGACAGATATTGGGCCGTGTCAAGGG<br>CTCTGGAGTATAATAGCAAGCGGACACCTAGGAGGATCAAGTGCATAATCCTTACAG<br>TCTGGCTCATAGCGGCAGTAATAAGCCTGCCCCCCCTTATATACAAAGGAGATCAGG<br>GCCCTCAACCCAGAGGGCGGCCACAATGCAAGCTGAATCAAGAGGCTTGGTACATTT<br>TGGCGAGCAGCATCGGAAGCTTCTTTGCCCCATGCCTGATCATGATTTTGGTATACCT<br>CAGGATTTACCTGATCGCTAAGCGGTCTAATAGACGCGGGCCTCGGGCTAAGGGAGG<br>ACCAGGCCAGGGGGAATCCAAGCAGCCTAGGCCTGATCACGGCGGCGCACTTGCCA<br>GTGCCAAGCTGCCCGCACTGGCCTCCGTCGCGTCAGCAAGAGAAGTCAATGGCCACA<br>GTAAATCTACTGGTGAAAAGGAAGAGGGCGAGACCCCTGAAGATACCGGAACCCGC<br>GCACTGCCTCCAAGTTGGGCTGCGCTGCCAAACTCTGGGCAGGGACAGAAGGAGGG<br>GGTGTGCGGCGCAAGTCCCGAGGACGAGGCCGAGGAGGAGGAAGAAGAAGAGGAG<br>GAAGAGGAGGAATGCGAGCCTCAAGCAGTGCCTGTAAGTCCAGCCTCTGCATGCTCA<br>CCTCCTCTTCAGCAGCCACAGGGCTCCAGAGTCCTCGCTACCCTCAGAGGGCAAGTG<br>CTGCTCGGAAGGGGTGTCGGTGCCATTGGAGGGCAATGGTGGCGCCGGCGGGCACA<br>GCTGACCAGAGAAAAGCGCTTCACCTTTGTGCTCGCCGTCGTGATCGGCGTATTCGTT<br>CTCTGCTGGTTCCCATTTTTCTTCTCCTACTCCTTGGGCGCGATTTGTCCTAAGCATTG<br>CAAAGTACCACATGGGCTCTTTCAATTTTTTTTCTGGATTGGTTATTGCAATAGCTCA<br>CTGAATCCTGTGATTTACACCATTTTCAATCAGGATTTCCGGCGGGCCTTTAGACGCA<br>TTCTGTGCAGGCCGTGGACCCAGACTGCATGGTAA
CHRM3 Δil3	P20309	Yes	ATGACGCTGCATAATAATTCAACCACAAGCCCTCTGTTCCCTAACATCAGTAGTAGCT<br>GGATACACTCTCCTAGCGACGCCGGACTTCCTCCCGGAACCGTGACCCACTTTGGGT<br>CATATAACGTCTCTCGGGCCGCAGGCAATTTTTCTTCCCCAGATGGGACAACCGACG<br>ATCCGCTCGGCGGCCACACTGTATGGCAGGTGGTTTTCATAGCCTTTCTGACCGGCAT<br>TCTGGCTCTGGTCACAATCATCGGCAATATTCTGGTAATTGTGTCCTTCAAGGTAAAC<br>AAACAACTGAAGACTGTCAATAACTACTTTCTGCTTAGTTTGGCATGCGCCGATCTGA<br>TTATCGGAGTGATAAGTATGAATCTCTTCACTACTTACATCATTATGAACAGATGGGC<br>TCTGGGTAACTTGGCTTGCGACCTGTGGCTCGCAATTGACTATGTCGCAAGCAACGC<br>CAGTGTGATGAACCTTCTGGTTATTAGCTTCGACCGCTATTTTAGCATTACACGCCCT<br>CTGACCTACAGAGCGAAACGCACTACAAAGCGGGCAGGAGTTATGATCGGCCTGGC<br>CTGGGTCATTTCCTTCGTGCTCTGGGCACCAGCCATCTTGTTCTGGCAGTATTTCGTC<br>GGTAAGAGGACAGTCCCACCAGGCGAGTGCTTCATCCAATTTTTGTCCGAGCCAACC<br>ATAACATTCGGCACAGCCATCGCGGCATTTTACATGCCAGTGACTATCATGACCATTC<br>TGTACTGGAGAATCTACAAAGAAACCGAAAAGCGGACAAAGGAGTTGGCAGGGCTT<br>CAGGCATCAGGCACAGAGACCCGGTCACAGATAACCAAGCGCAAGCGAATGTCACT<br>CGTGAAAGAAAAAAAAAGCAGCACAGACATTGAGCGCCATCTTGCTCGCTTTTATCAT<br>AACATGGACACCCTACAATATCATGGTCCTCGTGAACACTTTCTGCGACTCCTGTATT<br>CCTAAGACTTTCTGGAACCTCGGCTACTGGCTGTGCTATATCAACAGTACAGTTAACC<br>CTGTGTGCTACGCTCTCTGTAACAAGACCTTTAGGACCACGTTCAAGATGCTCCTCCT<br>GTGCCAGTGTGACAAAAAGAAACGACGAAAGCAGCAATATCAGCAGCGACAGAGTG<br>TGATCTTTCATAAACGGGCGCCAGAACAAGCGCTCTAA

Continued on next page

Table S7: Genes used in this study (Continued)

OPRM1	P35372-1	No	ATGGACAGCAGCGCTGCCCCCACGAACGCCAGCAATTGCACTGATGCCTTGGCGTAC<br>TCAAGTTGCTCCCCAGCACCCAGCCCCGGTTCCTGGGTCAACTTGTCCCACTTAGATG<br>GCAACCTGTCCGACCCATGCGGTCCGAACCGCACCGACCTGGGCGGGAGAGACAGC<br>CTGTGCCCTCCGACCGGCAGTCCCTCCATGATCACGGCCATCACGATCATGGCCCTCT<br>ACTCCATCGTGTGCGTGGTGGGGCTCTTCGGAAACTTCCTGGTCATGTATGTGATTGT<br>CAGATACACCAAGATGAAGACTGCCACCAACATCTACATTTTCAACCTTGCTCTGGC<br>AGATGCCTTAGCCACCAGTACCCTGCCCTTCCAGAGTGTGAATTACCTAATGGGAAC<br>ATGGCCATTTGGAACCATCCTTTGCAAGATAGTGATCTCCATAGATTACTATAACATG<br>TTCACCAGCATATTCACCCTCTGCACCATGAGTGTTGATCGATACATTGCAGTCTGCC<br>ACCCTGTCAAGGCCTTAGATTTCCGTACTCCCCGAAATGCCAAAATTATCAATGTCTG<br>CAACTGGATTCTCTCTTCAGCCATTGGTCTTCCTGTAATGTTCATGGCTACAACAAAA<br>TACAGGCAAGGTTCCATAGATTGTACACTAACATTCTCTCATCCAACCTGGTACTGGG<br>AAAACCTGCTGAAGATCTGTGTTTTCATCTTCGCCTTCATTATGCCAGTGCTCATCATT<br>ACCGTGTGCTATGGACTGATGATCTTGCGCCTCAAGAGTGTCCGCATGCTCTCTGGCT<br>CCAAAGAAAAGGACAGGAATCTTCGAAGGATCACCAGGATGGTGCTGGTGGTGGTG<br>GCTGTGTTCATCGTCTGCTGGACTCCCATTCACATTTACGTCATCATTAAAGCCTTGG<br>TTACAATCCCAGAAACTACGTTCCAGACTGTTTCTTGGCACTTCTGCATTGCTCTAGG<br>TTACACAAACAGCTGCCTCAACCCAGTCCTTTATGCATTTCTGGATGAAAACTTCAAA<br>CGATGCTTCAGAGAGTTCTGTATCCCAACCTCTTCCAACATTGAGCAACAAAACTCC<br>ACTCGAATTCGTCAGAACACTAGAGACCACCCCTCCACGGCCAATACAGTGGATAGA<br>ACTAATCATCAGCTAGAAAATCTGGAAGCAGAAACTGCTCCGTTGCCCTAA
MTNR1A	P48039	Yes	ATGCAAGGTAATGGTTCTGCTTTGCCAAATGCTTCTCAACCAGTTTTGAGAGGTGATG<br>GTGCTAGACCTTCTTGGTTGGCTTCTGCTTTAGCTTGTGTTTTGATTTTCACCATCGTT<br>GTCGATATCTTGGGTAACTTGTTGGTTATCTTGTCCGTCTACCGTAACAAGAAATTGA<br>GAAACGCTGGTAACATCTTCGTTGTTTCTTTGGCTGTTGCTGATTTGGTTGTTGCTATC<br>TATCCATATCCACTGGTCTTGATGTCCATTTTTAACAACGGTTGGAACTTGGGTTACT<br>TGCATTGTCAAGTTTCTGGTTTCTTGATGGGTTTGTCCGTTATTGGTTCCATTTTCAAC<br>ATTACCGGTATCGCCATTAACAGGTACTGTTACATTTGCCACTCACTGAAGTACGACA<br>AGTTGTACTCTTCTAAGAACTCCTTGTGCTACGTTTTGTTGATCTGGTTGTTAACTTTG<br>GCTGCTGTTTTGCCTAATTTGAGAGCTGGTACATTGCAATACGATCCAAGAATCTACT<br>CTTGTACCTTCGCTCAATCTGTTTCTTCTGCTTACACTATTGCCGTTGTCGTTTTCCATT<br>TTTTGGTCCCAATGATTATCGTCATCTTCTGCTACTTGAGAATCTGGATTTTGGTCTTG<br>CAAGTCAGACAAAGAGTTAAGCCAGATAGAAAGCCAAAATTGAAGCCACAAGACTT<br>CAGAAACTTCGTTACCATGTTTGTGGTTTTCGTTTTGTTCGCTATTTGTTGGGCTCCAT<br>TGAACTTTATTGGTTTGGCAGTTGCTTCTGATCCAGCTTCTATGGTTCCAAGAATTCC<br>AGAATGGTTGTTCGTTGCTTCTTACTACATGGCTTACTTCAACTCTTGTTTGAACGCCA<br>TTATCTACGGCTTGTTGAATCAGAACTTTAGGAAAGAGTACAGGCGTATCATCGTTTC<br>TTTGTGTACTGCTAGAGTTTTCTTCGTCGATTCCTCTAATGATGTTGCCGATAGAGTTA<br>AGTGGAAACCATCTCCATTGATGACCAACAACAATGTTGTCAAGGTTGACTCCGTTG<br>GATCCTGA
NanoLuc	(*62*)	No	ATGGTCTTCACACTCGAAGATTTCGTTGGGGACTGGCGACAGACAGCCGGCTACAAC<br>CTGGACCAAGTCCTTGAACAGGGAGGTGTGTCCAGTTTGTTTCAGAATCTCGGGGTG<br>TCCGTAACTCCGATCCAAAGGATTGTCCTGAGCGGTGAAAATGGGCTGAAGATCGAC<br>ATCCATGTCATCATCCCGTATGAAGGTCTGAGCGGCGACCAAATGGGCCAGATCGAA<br>AAAATTTTTAAGGTGGTGTACCCTGTGGATGATCATCACTTTAAGGTGATCCTGCACT<br>ATGGCACACTGGTAATCGACGGGGTTACGCCGAACATGATCGACTATTTCGGACGGC<br>CGTATGAAGGCATCGCCGTGTTCGACGGCAAAAAGATCACTGTAACAGGGACCCTGT<br>GGAACGGCAACAAAATTATCGACGAGCGCCTGATCAACCCCGACGGCTCCCTGCTGT<br>TCCGAGTAACCATCAACGGAGTGACCGGCTGGCGGCTGTGCGAACGCATTCTGGCGT<br>AA

Table S8: Yeast strains utilised in this study, ScFH236 and 237 are based on design 4 presented in Shaw et al 2019 (*63*)

ScFH236	BY4741-MATa,sst2Δ0,far1Δ0,bar1Δ0,ste2Δ0,ste12Δ0,gpa1Δ0,ste3Δ0,mf(alpha)1Δ0,mf(alpha)2Δ0,mfa1Δ0,mfa2Δ0,gpr1Δ0,gpa2Δ0,LexO(6x)-pLEU2m-NanoLuc-tTDH1-pCCW12-STE2-tSSA1-pPGK1-GPA1-YIGLC-tENO2-pRAD27-LexA-PRD-tENO1-URA3	This study
ScFH237	BY4741-MATa,sst2Δ0,far1Δ0,bar1Δ0,ste2Δ0,ste12Δ0,gpa1Δ0,ste3Δ0,mf(alpha)1Δ0,mf(alpha)2Δ0,mfa1Δ0,mfa2Δ0,gpr1Δ0,gpa2Δ0,LexO(6x)-pLEU2m-NanoLuc-tTDH1-pCCW12-STE2-tSSA1-pPGK1-GPA1-YIGLC-tENO2-pRAD27-LexA-PRD-tENO1-URA3	This study
ScS1	ScFH237 + PCCW12-HsHTR1A-TCYC1	This study
ScS2	ScFH237 + PCCW12-HsHTR4-TCYC1	This study
ScS3	ScFH237 + PCCW12-HsADRA2A-TCYC1	This study
ScS4	ScFH236 + PCCW12-HsADRA2B-TCYC1	This study
ScS5	ScFH237 + PCCW12-OPRM1-TCYC1	This study
ScS6	ScFH237 + PCCW12-HsCHRM3-TCYC1	This study
ScS7	ScFH237 + PCCW12-HsMTNR1A-TCYC1	This study

Table S9: **Selected Subset of pdCSM Dataset**. hGPCRs were selected which also occurred in the set of 128 hGPCRs investigated in this paper. Number of training (47013) and test (7114) data points are summarised.

Target hGPCR	Test Entries	Train Entries
5HTR2C (P28335)	353	2765
5HTR6 (P50406)	345	2699
ADORA1A (P30542)	424	3409
ADORA2B (P29275)	274	1835
ADORA3 (P0DMS8)	413	3100
ADRA1A (P35348)	253	1645
BDKRB1 (P46663)	148	608
CCR3 (P51677)	184	947
CHRM3 (P20309)	310	1698
CHRM4 (P08173)	141	837
CHRM5 (P08912)	139	820
DRD4 (P21917)	276	2059
EDNRB (P24530)	172	815
GNRHR (P30968)	276	1097
GPBAR1 (Q8TDU6)	71	372
GPR35 (Q9HC97)	72	408
GPR44 (Q9Y5Y4)	342	2407
HCAR2 (Q8TDS4)	70	434
HRH3 (Q9Y5N1)	464	3133
HTR1A (P08908)	420	3370
MCHR1 (Q99705)	435	3286
MTNR1A (P48039)	152	891
NK2R (P21452)	160	762
OPRM1 (P35372)	624	4651
P2RY1 (P47900)	107	461
PTGER1 (P34995)	101	640
S1PR3 (Q99500)	149	939
S1PR5 (Q9H228)	68	349
SSTR5 (P35346)	171	576

Table S10: **Addressing similarities and differences of CSNN with published methods**. We highlight that most published methods are compound-to-prediction architectures (which answer the query P(y|c,t)), while CSNNs are distinctly different due to their neighbourhood-to-prediction architecture (P(y|c,t,$\mathcal{N}$(c))).

**Article**	**Prediction Objective**	**Model Architecture**	**Difference**	**Similarity**	**Notes**
Lim et al. J. Chem. Inf. Model. 2019, 59, 9, 3981–3988	Classification (bind/not bind) P(y|t,c)	GAT	Require explicit 3D binding poses for training. Input for inference is an explicit 3D pose. Works only on a single ligand-target pose during inference.	Use GAT architecture.	Distantly related. Not directly comparable.
Jiang et al., RSC Adv, 2020, 10:20701-20712	Regression (DTA), P(y|t,c)	GCN and GAT	Target is represented as a contact map, which carries far less information than the ESM embeddings. They predict drug binding affinity. Not directly developed for GPCRs. They operate only on a single drug-target pair during inference.	Addresses drug-target affinity (DTA), which our extended experiments also address. Graph neural network usage.	Conceptually dissimilar, does not use chemical neighborhoods, chemical space networks, or message passing at this level.
Bguyen et al., Bioinformatics, 2021, 37(8): 1140-1147	Regression (DTA), P(y|t,c)	GNNs (GIN, GAT, GCN)	We kindly stress again that they operate only on a single drug-target pair during inference. Uses one-hot encoding of sequence, which is a very sparse representation.	GIN, GAT, GNN usage. Ibid. as above with DTA.	Method cannot integrate available data dynamically during inference
Sun et al., Bioinformatics, 2024, 40(3): btae135	Classification (bind/not bind) P(y|t,c)	GNN (to integrate information from concatenated pre-trained representations)	Uses handcrafted descriptors/featurization (which are always outperformed by learned representations), but they operate only on a single drug-target pair during inference.	Adresses DTI and uses GNNs. ESM is used. Pre-trained Chemformer is used for a learned representation.	Method cannot integrate available data dynamically during inference.
Velloso et al. Bioinformatics Advances, Volume 1, Issue 1, 2021, vbab031	Regression (DTA), P(y|c) (one model per target)	RF, Gradient Boosting.	Use handcrafted descriptors and simple ML models (XGboost, RF). Again, we kindly point out that they operate only on a single drug-target pair during inference. The repeated occurrence of this seems to indicate that a misunderstanding of the paper has occurred.	GPCR focus, DTA. They, however, build one model per target rather than a single model (their method doesn't enable this either without the addition of extremely sparse OHE or ESM-2 embeddings).	We have benchmarked against their data, as it is well documented and has available test-train splits.
Kanai et al., Molecules. 2021 ;26(17):5131	Classification (bind/not bind) P(y|t,c)	SVM	The tensor product of compound and protein is an elegant approach to model their joint space. However, they operate only on a single drug-target pair during inference. Although this paper is the most relevant suggestion so far.	Uses a similarity metric, with a kernel, to make binary activity predictions with a SVM. This is partly related as they implicitly model neighbourhoods in the tensor product space with a decision boundary.	Interesting use of kernel based method to look at similarities between compounds and GPCR targets.

Table S10: **Addressing similarities and differences of CSNN with published methods**. We highlight that most published methods are compound-to-prediction architectures (which answer the query P(y|c,t)), while CSNNs are distinctly different due to their neighbourhood-to-prediction architecture (P(y|c,t,$\mathcal{N}$(c))).

Article	Prediction Objective	Model Architecture	Difference	Similarity	Notes
Cai et al. J Chem Inf Model. 2021 Apr 26;61(4):1570-1582	P(y|t,c), binary classification (active, inactive).	Distilled sequence alignment embedding (DISAE, fine-tuned) and pre-trained MPNN for compound.	Use only a single compound-target pair during training and inference.	GPCR focus and classification objective.	
Lu et al. IEEE J Biomed Health Inform. 2023 doi: 10.1109/ JBHI.2023. 3307928	Both classification and regression P(y|t,c)	CNN	We stress again the fact that they operate only on a single drug-target pair during inference. Fig 5. Shows that in fact their correlations are neither strong nor good predictions. Model shows strong 'mode seeking' behavior, which our benchmark on DTA does not. Perhaps a point which applies to many of the aforementioned papers is also the lack of experimental validation.	GPCR focus.	
Ahmed et al. Sci Rep 11, 9510 (2021)	Classification (bind/not bind) P(y|t,c)	RF/ XG-Boost / SVMs	They operate only on a single drug-target pair during inference. No experimental verification. Use simple algorithmic descriptors from RDkit, which since the paper in 2019 have strong support for always being outperformed by learned representations: doi.org/10.1021/ acs.jcim.9b00237	GPCR focus. We in fact use their hyperparameter searches to guide ours, thus allowing for a comparison with published DTI approaches. As we show in Figure 3C, RF/MLP methods performs markedly worse in comparison to CSNN or argmax().	The GPCR_LigandClassify.py paper. Their test/train data is not available nor reproducible.
Zhang et al. Briefings in Bioinformatics, Volume 25, Issue 4, July 2024, bbae281	Both classification and regression P(y|t,c)	GNN	We point out again that they operate only on a single drug-target pair during inference. Single model per target, no way of doing one model for all targets. AUC discriminatory values are ≈ 0.72 (0.48-0.93) (AUC = 0.5). Average pearson correlation is 0.39, again showing far lower performance.	GPCR focus. Perform experimental verification.	
Cai T, et al.. Bioinformatics ;38(9):2561-2570	Classification (bind/not bind) P(y|t,c) and bioactivity class (antagonist / agonist).	Unclear (Use of several pre-trained models).	Out of distribution testing, which is quite elegant. OOD for proteins split and chemical similarity split. We kindly stress the fact that the query (P(y|t,c)), is unlike CSNN. The method operates only on a single drug-target pair during inference. Fig 5. Training curves indicate significant difficulty in training (high variance) and the OOD training curves barely show any changes in the precision (nothing learned by model). No experimental validation.	Data sources are quite similar.	
Huang et al. J Cheminform 16, 10 (2024). doi.org/ 10.1186/ s13321-024-00806-3	P(y|t,c) in the Regression setting (EC50)	CatBoost, RF, and LightGBM	Uses rule-based embedding (for both sequence and compound) and concatenate them, thus only allow for prediction of a single drug-target interaction, without using available data during inference. This is a compound-to-prediction architecture unlike CSNN.	GPCR focus. Similar data-type.	Does not deal with using available data during inference.

Table S11: **HTS-Screen compliance table**. The data in this table relates to the performed yeast based HTS

Category	Parameter	Description
Assay	Type of assay	Cell-based
	Targets	Q13639, P35372, P08913, P18089<br>P48039, P08908, P20309
	Primary measurement	Relative increase in luminescence
	Key reagents	Promega nano-glo substrate (N1110)<br>and Merck Cell lytic Y
	Assay protocol	Methods: Bioactivity assays
Library	Library size	539 compounds
	Library composition	Small molecules, 390 FDA/EMA<br>approved drugs and 149<br>plant natural products containing<br>at least 1 indole ring moeity
	Source	Prestwick and Chemfaces
Screen	Format	Small volume 96-well plates<br>(Greiner 675083)
	Concentration(s) tested	10μM compound 1% DMSO
	Plate controls	3
	Detection instrument and software	SynergyMX plate reader, with MD Gen5
	Assay validation/QC	SD of controls <10%
Post-HTS analysis	Hit criteria	Z score >3
	Additional comments	Complete screen data is available<br>at the Zenodo archive<br>associated with this paper<br>(CSNN\02_DTU_dataprocessing.zip\<br>02_DTU_dataprocessing\<br>data_input\001_screen_pruned.csv)

a) Extended Table for Novel Hits.

Name	Use [Approved]	Z-score [novel]	Predicted Label	Predicted -log10(Ki)	Z-score [confirmed]
Tinoridine	Against pain and inflammation	HTR4: 5.87	OPRM1: No Effect<br>CHRM3: No Effect	OPRM1: 4.113<br>CHRM3: 6.630	OPRM1: 0.068<br>CHRM3: -0.553
Piribidil	Parkinsons disease	ADRA2B: 4.68	OPRM1: No Effect<br>HTR1A: Antagonist<br>HTR1A (argmax): Partial Agonist	OPRM: 4.042<br>HTR1A: 5.656	OPRM1: 0.336<br>HTR1A: 0.747

Table S12: Extended Table for Figure 4g, which summarises also DTIs for which predictions were produced (data in the chemical neighbourhood was available). We see that "No Effect" label predictions are reflected in the non-statically significant Z-score. Piribidil has a label disagreement from the argmax prediction and the NN_θ^6 model and shows a non-statistically significant Z-score as well as low predicted K_i values (from RF + N model).

S11 Supplementary References

1. H. Matter, C. Buning, D. D. Stefanescu, S. Ruf, G. Hessler, Using Graph Databases to Investigate Trends in Structure–Activity Relationship Networks. *J. Chem. Inf. Model.* **60**, 6120–6134 (2020).
2. D. Stumpfe, H. Hu, J. Bajorath, Evolving Concept of Activity Cliffs. *ACS Omega* **4**, Publisher: American Chemical Society, 14360–14368, (2024; `https://doi.org/10.1021/acsomega.9b02221`) (2019).
3. R. Kunimoto, J. Bajorath, Combining Similarity Searching and Network Analysis for the Identification of Active Compounds. *ACS Omega* **3**, 3768–3777 (2018).
4. N. Amoroso, N. Gambacorta, F. Mastrolorito, M. V. Togo, D. Trisciuzzi, A. Monaco, E. Pantaleo, C. D. Altomare, F. Ciriaco, O. Nicolotti, Making sense of chemical space network shows signs of criticality. *Scientific Reports* (2023).
5. J. Yi, S. Lee, S. Lim, C. Cho, Y. Piao, M. Yeo, D. Kim, S. Kim, S. Lee, Exploring chemical space for lead identification by propagating on chemical similarity network. *Computational and Structural Biotechnology Journal* **21**, 4187–4195 (2023).
6. W. Fan, S. Wang, J. Huang, Z. Chen, Y. Song, W. Tang, H. Mao, H. Liu, X. Liu, D. Yin, Q. Li, *Graph Machine Learning in the Era of Large Language Models (LLMs)*, 2024, (`http://arxiv.org/abs/2404.14928`).
7. P. Chandak, K. Huang, M. Zitnik, Building a knowledge graph to enable precision medicine. en, *Sci Data* **10**, Publisher: Nature Publishing Group, 67, ISSN: 2052-4463, (2024; `https://www.nature.com/articles/s41597-023-01960-3`) (2023).
8. A. Santos, A. R. Colaço, A. B. Nielsen, L. Niu, M. Strauss, P. E. Geyer, F. Coscia, N. J. W. Albrechtsen, F. Mundt, L. J. Jensen, M. Mann, A knowledge graph to interpret clinical proteomics data. *Nat Biotechnol* **40**, 692–702, ISSN: 1546-1696 (2022).
9. R. T. Sousa, S. Silva, C. Pesquita, Explaining protein–protein interactions with knowledge graph-based semantic similarity. *Computers in Biology and Medicine* **170**, 108076, ISSN: 0010-4825 (2024).
10. Z. Lyu, Y. Wu, J. Lai, M. Yang, C. Li, W. Zhou, Knowledge Enhanced Graph Neural Networks for Explainable Recommendation. *IEEE Transactions on Knowledge and Data Engineering* **35**, 4954–4968, ISSN: 1558-2191 (2023).
11. L. Werner, N. Layaïda, P. Genevès, S. Chlyah, *Knowledge Enhanced Graph Neural Networks for Graph Completion*, 2023.
12. R. Sato, *Training-free Graph Neural Networks and the Power of Labels as Features*, 2024, arXiv: `2404.19288 (cs.LG)`, (`https://arxiv.org/abs/2404.19288`).
13. J. Jiménez-Luna, F. Grisoni, G. Schneider, Drug discovery with explainable artificial intelligence. *Nat Mach Intell* **2**, 573–584 (2020).
14. T. Beckers, *An Introduction to Gaussian Process Models*, 2021, (2024; `http://arxiv.org/abs/2102.05497`).
15. A. de Falco, Z. Dezso, F. Ceccarelli, L. Cerulo, A. Ciaramella, M. Ceccarelli, Adaptive one-class Gaussian processes allow accurate prioritization of oncology drug targets. *Bioinformatics* **37**, 1420–1427, ISSN: 1367-4803 (2021).
16. T. van Laarhoven, S. B. Nabuurs, E. Marchiori, Gaussian interaction profile kernels for predicting drug–target interaction. *Bioinformatics* **27**, 3036–3043, ISSN: 1367-4803 (2011).
17. M. Fontana, G. Zeni, S. Vantini, Conformal Prediction: a Unified Review of Theory and New Challenges. *arXiv* **29**, ISSN: 1350-7265 (2023).
18. S. Arvidsson McShane, U. Norinder, J. Alvarsson, E. Ahlberg, L. Carlsson, O. Spjuth, CPSign: conformal prediction for cheminformatics modeling. *Journal of Cheminformatics* **16**, 75, ISSN: 1758-2946 (2024).
19. S. Kim, J. Chen, T. Cheng, A. Gindulyte, J. He, S. He, Q. Li, B. A. Shoemaker, P. A. Thiessen, B. Yu, L. Zaslavsky, J. Zhang, E. E. Bolton, PubChem 2023 update. *Nucleic Acids Res.* **51**, D1373–D1380 (2023).
20. P.-K. Li, G.-H. Chu, M. L. Gillen, T. Parekh, P. A. Witt-Enderby, The development of a charged melatonin receptor ligand. *Bioorg. Med. Chem. Lett.* **7**, 2409–2414 (1997).
21. J. Saxena, F. Nicolini, M. Lockley, Abstract 3801: Reversing chemoresistance in high grade serous ovarian cancer. *Cancer Res.* **79**, 3801–3801 (2019).
22. N. J. Kapolka, G. J. Taghon, J. B. Rowe, W. M. Morgan, J. F. Enten, N. A. Lambert, D. G. Isom, DCyFIR: a high-throughput CRISPR platform for multiplexed G protein-coupled receptor profiling and ligand discovery. *Proc. Natl. Acad. Sci. U. S. A.* **117**, 13117–13126 (2020).
23. E. D. Jensen, M. Deichmann, X. Ma, R. U. Vilandt, G. Schiesaro, M. B. Rojek, B. Lengger, L. Eliasson, J. M. Vento, D. Durmusoglu, S. P. Hovmand, I. Al'Abri, J. Zhang, N. Crook, M. K. Jensen, Engineered cell differentiation and sexual reproduction in probiotic and mating yeasts. *Nat. Commun.* **13**, 6201 (2022).
24. A. Vardanian, *SimSIMD*, github.com/ashvardanian/SimSIMD, 2023.

25. V. F. Scalfani, V. D. Patel, A. M. Fernandez, Visualizing chemical space networks with RDKit and NetworkX. *Journal of Cheminformatics* **14**, 87 (2022).

26. J. P. L. Velloso, D. B. Ascher, D. E. V. Pires, pdCSM-GPCR: predicting potent GPCR ligands with graph-based signatures. *Bioinformatics Advances* **1**, ISSN: 2635-0041 (2021).

27. B. Zdrazil, E. Felix, F. Hunter, E. J. Manners, J. Blackshaw, S. Corbett, M. de Veij, H. Ioannidis, D. M. Lopez, J. F. Mosquera, M. P. Magarinos, N. Bosc, R. Arcila, T. Kizilören, A. Gaulton, A. P. Bento, M. F. Adasme, P. Monecke, G. A. Landrum, A. R. Leach, The ChEMBL Database in 2023: a drug discovery platform spanning multiple bioactivity data types and time periods. *Nucleic Acids Research* **52**, D1180–D1192 (2024).

28. M. N. Davies, A. Secker, A. A. Freitas, M. Mendao, J. Timmis, D. R. Flower, On the hierarchical classification of G protein-coupled receptors. *Bioinformatics* **23**, 3113–3118 (2007).

29. L. van der Maaten, G. Hinton, Visualizing Data using t-SNE. *Journal of Machine Learning Research* **9**, 2579–2605 (2008).

30. D. Probst, J.-L. Reymond, Visualization of very large high-dimensional data sets as minimum spanning trees. *J. Cheminform.* **12**, 12 (2020).

31. Z. Wu, W. Li, G. Liu, Y. Tang, Network-Based Methods for Prediction of Drug-Target Interactions. *Frontiers in Pharmacology* **9**, ISSN: 1663-9812 (2018).

32. J. M. Remington, K. T. McKay, N. B. Beckage, J. B. Ferrell, S. T. Schneebeli, J. Li, GPCRLigNet: rapid screening for GPCR active ligands using machine learning. *Journal of Computer-Aided Molecular Design* **37**, 147–156, ISSN: 15734951 (3 2023).

33. K. Amara, R. Rodríguez-Pérez, J. Jiménez-Luna, Explaining compound activity predictions with a substructure-aware loss for graph neural networks. *Journal of Cheminformatics* **15**, 67 (2023).

34. Z. Wu, H. Ma, Z. Liu, L. Zheng, Z. Yu, S. Cao, W. Fang, L. Wu, W. Li, G. Liu, J. Huang, Y. Tang, wSDTNBI: a novel network-based inference method for virtual screening. *Chem. Sci.* **13**, 1060–1079 (2022).

35. Z. Yu, Z. Wu, W. Li, G. Liu, Y. Tang, ADENet: a novel network-based inference method for prediction of drug adverse events. *Brief. Bioinform.* **23** (2022).

36. F. Fiorino, B. Severino, E. Magli, A. Ciano, G. Caliendo, V. Santagada, F. Frecentese, E. Perissutti, 5-HT(1A) receptor: an old target as a new attractive tool in drug discovery from central nervous system to cancer. *J. Med. Chem.* **57**, 4407–4426 (2014).

37. E. J. Jeong, S. Y. Chung, H. N. Hong, S.-W. Oh, J. Y. Sim, The novel, potent and highly selective 5-HT4 receptor agonist YH12852 significantly improves both upper and lower gastrointestinal motility. *Br. J. Pharmacol.* **175**, 485–500 (2018).

38. K. Köhler-Forsberg, B. Ozenne, S. V. Larsen, A. S. Poulsen, E. B. Landman, V. H. Dam, C.-T. Ip, A. Jørgensen, C. Svarer, G. M. Knudsen, V. G. Frokjaer, M. B. Jørgensen, Concurrent anxiety in patients with major depression and cerebral serotonin 4 receptor binding. A NeuroPharm-1 study. *Transl. Psychiatry* **12**, 273 (2022).

39. M. Ono, E. Takamura, K. Shinozaki, T. Tsumura, T. Hamano, Y. Yagi, K. Tsubota, Therapeutic effect of cevimeline on dry eye in patients with Sjögren's syndrome: a randomized, double-blind clinical study. *Am. J. Ophthalmol.* **138**, 6–17 (2004).

40. A. W. Cowley Jr, Chrm3 Gene and M3 Muscarinic Receptors Contribute to Salt-Sensitive Hypertension. *Hypertension* **72**, 588–591 (2018).

41. S. P. Moran, J. Maksymetz, P. J. Conn, Targeting Muscarinic Acetylcholine Receptors for the Treatment of Psychiatric and Neurological Disorders. *Trends Pharmacol. Sci.* **40**, 1006–1020 (2019).

42. A. K. Paul, C. M. Smith, M. Rahmatullah, V. Nissapatorn, P. Wilairatana, M. Spetea, N. Gueven, N. Dietis, Opioid Analgesia and Opioid-Induced Adverse Effects: A Review. *Pharmaceuticals* **14** (2021).

43. S. Rivara, M. Mor, A. Bedini, G. Spadoni, G. Tarzia, Melatonin receptor agonists: SAR and applications to the treatment of sleep-wake disorders. *Curr. Top. Med. Chem.* **8**, 954–968 (2008).

44. Z. P. Khan, C. N. Ferguson, R. M. Jones, alpha-2 and imidazoline receptor agonists. Their pharmacology and therapeutic role. *Anaesthesia* **54**, 146–165 (1999).

45. T. Wang, Z. Li, M. E. Cvijic, L. Zhang, C. S. Sum, *Measurement Of cAMP For Gαs- And Gαi Protein-Coupled Receptors (GPCRs)* (Eli Lilly & Company and the National Center for Advancing Translational Sciences, Bethesda (MD), 2017).

46. C. Bergsdorf, C. Kropp-Goerkis, I. Kaehler, L. Ketscher, U. Boemer, K. Parczyk, B. Bader, A one-day, dispense-only IP-One HTRF assay for high-throughput screening of Galphaq protein-coupled receptors: towards cells as reagents. *Assay Drug Dev. Technol.* **6**, 39–53 (2008).

47. Q. Ma, L. Ye, H. Liu, Y. Shi, N. Zhou, An overview of Ca2+ mobilization assays in GPCR drug discovery. *Expert Opin. Drug Discov.* **12**, 511–523 (2017).

48. R. H. Oakley, C. C. Hudson, R. D. Cruickshank, D. M. Meyers, R. E. Payne Jr, S. M. Rhem, C. R. Loomis, The cellular distribution of fluorescently labeled arrestins provides a robust, sensitive, and universal assay for screening G protein-coupled receptors. *Assay Drug Dev. Technol.* **1**, 21–30 (2002).

49. J. Wang, X. Xie, Development of a quantitative, cell-based, high-content screening assay for epidermal growth factor receptor modulators. *Acta Pharmacol. Sin.* **28**, 1698–1704 (2007).

50. P. G. Strange, Use of the GTPγS ([35S]GTPγS and Eu-GTPγS) binding assay for analysis of ligand potency and efficacy at G protein-coupled receptors. *Br. J. Pharmacol.* **161**, 1238–1249 (2010).

51. D. B. Bylund, J. D. Deupree, M. L. Toews, Radioligand-binding methods for membrane preparations and intact cells. *Methods Mol. Biol.* **259**, 1–28 (2004).

52. D. B. Bylund, M. L. Toews, Radioligand binding methods: practical guide and tips. *Am. J. Physiol.* **265**, L421–9 (1993).

53. H. Chen, C. E. Rosen, J. A. González-Hernández, D. Song, J. Potempa, A. M. Ring, N. W. Palm, Highly multiplexed bioactivity screening reveals human and microbiota metabolome-GPCRome interactions. *Cell* **186**, 3095–3110 (2023).

54. K. Miettinen, N. Leelahakorn, A. Almeida, Y. Zhao, L. R. Hansen, I. E. Nikolajsen, J. B. Andersen, M. Givskov, D. Staerk, S. Bak, S. C. Kampranis, A GPCR-based yeast biosensor for biomedical, biotechnological, and point-of-use cannabinoid determination. *Nat. Commun.* **13**, 3664 (2022).

55. J. Vucicevic, T. Srdic-Rajic, M. Pieroni, J. M. M. Laurila, V. Perovic, S. Tassini, E. Azzali, G. Costantino, S. Glisic, D. Agbaba, M. Scheinin, K. Nikolic, M. Radi, N. Veljkovic, A combined ligand- and structure-based approach for the identification of rilmenidine-derived compounds which synergize the antitumor effects of doxorubicin. *Bioorg. Med. Chem.* **24**, 3174–3183 (2016).

56. D. Mendez, A. Gaulton, A. P. Bento, J. Chambers, M. De Veij, E. Félix, M. P. Magariños, J. F. Mosquera, P. Mutowo, M. Nowotka, M. Gordillo-Marañón, F. Hunter, L. Junco, G. Mugumbate, M. Rodriguez-Lopez, F. Atkinson, N. Bosc, C. J. Radoux, A. Segura-Cabrera, A. Hersey, A. R. Leach, ChEMBL: towards direct deposition of bioassay data. *Nucleic acids research* **47**, D930–D940, ISSN: 0305-1048 (2019).

57. O. Russo, M. Cachard-Chastel, C. Rivière, M. Giner, J.-L. Soulier, M. Berthouze, T. Richard, J.-P. Monti, S. Sicsic, F. Lezoualc'h, I. Berque-Bestel, Design, synthesis, and biological evaluation of new 5-HT4 receptor agonists: application as amyloid cascade modulators and potential therapeutic utility in Alzheimer's disease. *J. Med. Chem.* **52**, 2214–2225 (2009).

58. J. S. D. Kumar, V. J. Majo, S.-C. Hsiung, M. S. Millak, K.-P. Liu, H. Tamir, J. Prabhakaran, N. R. Simpson, R. L. Van Heertum, J. J. Mann, R. V. Parsey, Synthesis and in vivo validation of [O-methyl-11C]2-{4-[4-(7-methoxynaphthalen-1-yl)piperazin- 1-yl]butyl}-4-methyl-2H-[1,2,4]triazine-3,5-dione: a novel 5-HT1A receptor agonist positron emission tomography ligand. *J. Med. Chem.* **49**, 125–134 (2006).

59. I. Fish, A. Stößel, K. Eitel, C. Valant, S. Albold, H. Huebner, D. Möller, M. J. Clark, R. K. Sunahara, A. Christopoulos, B. K. Shoichet, P. Gmeiner, Structure-Based Design and Discovery of New M2 Receptor Agonists. *J. Med. Chem.* **60**, 9239–9250 (2017).

60. G. Somalo-Barranco, C. Serra, D. Lyons, H. D. Piggins, R. Jockers, A. Llebaria, Design and Validation of the First Family of Photo-Activatable Ligands for Melatonin Receptors. *J. Med. Chem.* **65**, 11229–11240 (2022).

61. E. S. Gutman, E. Bow, F. Li, A. Sulima, S. Kaska, R. Crowley, T. E. Prisinzano, Y.-S. Lee, S. A. Hassan, G. H. Imler, J. R. Deschamps, A. E. Jacobson, K. C. Rice, G-Protein biased opioid agonists: 3-hydroxy-N-phenethyl-5-phenylmorphans with three-carbon chain substituents at C9. *RSC Med Chem* **11**, 896–904 (2020).

62. C. G. England, E. B. Ehlerding, W. Cai, NanoLuc: A Small Luciferase Is Brightening Up the Field of Bioluminescence. *Bioconjugate Chemistry* **27**, 1175–1187 (2016).

63. W. M. Shaw, H. Yamauchi, J. Mead, G.-O. F. Gowers, D. J. Bell, D. Öling, N. Larsson, M. Wigglesworth, G. Ladds, T. Ellis, Engineering a Model Cell for Rational Tuning of GPCR Signaling. *Cell* **177**, 782–796.e27 (2019).
