# Supplementary material for: Labels as a feature: Network homophily for systematically annotating human GPCR drug-target interactions
Source: Nat Commun. 2025 May 3;16:4121. doi: 10.1038/s41467-025-59418-6 (PMC12048553; doi:10.1038/s41467-025-59418-6)
Supplement: Supplementary file 2 — Description of Additional Supplementary Files [file 41467_2025_59418_MOESM2_ESM.pdf]

Description of Additional Supplementary Files:

Supplementary Data 1: A price estimation for running a 4000 datapoint assay on GPCR-ligand interactions. all prices listed in USD currency reported as of 01-03-2024
