# Supplementary material for: Labels as a feature: Network homophily for systematically annotating human GPCR drug-target interactions
Source: Nat Commun. 2025 May 3;16:4121. doi: 10.1038/s41467-025-59418-6 (PMC12048553; doi:10.1038/s41467-025-59418-6)
Supplement: Supplementary file 4 — Reporting Summary [file 41467_2025_59418_MOESM4_ESM.pdf]

nature portfolio

Corresponding author(s): Emil D. Jensen & Carlos G. Acevedo-Rocha

Last updated by author(s): Feb 25, 2025

Reporting Summary

Nature Portfolio wishes to improve the reproducibility of the work that we publish. This form provides structure for consistency and transparency in reporting. For further information on Nature Portfolio policies, see our Editorial Policies and the Editorial Policy Checklist.

Statistics

For all statistical analyses, confirm that the following items are present in the figure legend, table legend, main text, or Methods section.

n/a	Confirmed	
☐	☒	The exact sample size (n) for each experimental group/condition, given as a discrete number and unit of measurement
☐	☒	A statement on whether measurements were taken from distinct samples or whether the same sample was measured repeatedly
☐	☒	The statistical test(s) used AND whether they are one- or two-sided<br>*Only common tests should be described solely by name; describe more complex techniques in the Methods section.*
☐	☒	A description of all covariates tested
☐	☒	A description of any assumptions or corrections, such as tests of normality and adjustment for multiple comparisons
☐	☒	A full description of the statistical parameters including central tendency (e.g. means) or other basic estimates (e.g. regression coefficient) AND variation (e.g. standard deviation) or associated estimates of uncertainty (e.g. confidence intervals)
☐	☒	For null hypothesis testing, the test statistic (e.g. F, t, r) with confidence intervals, effect sizes, degrees of freedom and P value noted<br>*Give P values as exact values whenever suitable.*
☒	☐	For Bayesian analysis, information on the choice of priors and Markov chain Monte Carlo settings
☐	☒	For hierarchical and complex designs, identification of the appropriate level for tests and full reporting of outcomes
☐	☒	Estimates of effect sizes (e.g. Cohen's d, Pearson's r), indicating how they were calculated

Our web collection on statistics for biologists contains articles on many of the points above.

Software and code

Policy information about availability of computer code

Data collection	Data was sourced from the ChEMBL and IUPHAR repositories. For CHEMBL, the dataset was downloaded directly from the websites UI. (https://www.ebi.ac.uk/chembl/web_components/explore/compounds/) the version of ChEMBL is ChEMBL07 and used under license CC Attribution-ShareAlike 3.0 Unported license. For IUPHAR/BPS, the datasets were retrieved their API: https://www.guidetopharmacology.org/services/ under under CC Attribution-ShareAlike 4.0 International License<br>The pdCSM\cite{velloso_pdcsm-gpcr_2021} dataset was sourced from \url{https://biosig.lab.uq.edu.au/pdcsm_gpcr/} with CC-BY 4.0 license.<br>Our code for retrieval, datasets generated and scripts for analysis is made available at Zenodo CSNN DOI: 10.5281/zenodo.12532112 under Creative Commons Attribution 4.0 International license
Data analysis	Python 3.10.8, Data processing, training, evalutation, and inference scripts are custom written by the authors and provided in full.<br>The following list contains all packages and dependencies used in to generate CSNN:<br>Open-source (GPL-3.0 license) pre-trained model for compound representations https://github.com/cansyl/TransferLearning4DTI/.<br>- appnope=0.1.4 - asttokens=2.4.1 - bzip2=1.0.8 - ca-certificates=2024.6.2 - colorama=0.4.6 - comm=0.2.2 - debugpy=1.8.1 - decorator=5.1.1 - exceptiongroup=1.2.0 - executing=2.0.1 - filelock=3.15.1 - gmp=6.3.0 - gmpy2=2.1.5 - importlib-metadata=7.1.0 - importlib_metadata=7.1.0 - ipykernel=6.29.4 - ipython=8.25.0 - jedi=0.19.1 - jinja2=3.1.4 - jupyter_client=8.6.2 - jupyter_core=5.7.2 - krb5=1.21.2 - libblas=3.9.0 - libcblas=3.9.0 - libcxx=17.0.6 - libedit=3.1.20191231 - libffi=3.4.2 - libgfortran=5.0.0 - libgfortran5=13.2.0 - liblapack=3.9.0 - libopenblas=0.3.24 - libsodium=1.0.18 - libsqlite=3.46.0 - libzlib=1.3.1 - llvm-openmp=15.0.7 - markupsafe=2.1.5 - matplotlib-inline=0.1.7 - mpc=1.3.1 - mpfr=4.2.1 - mpmath=1.3.0 - ncurses=6.5 - nest-asyncio=1.6.0 - networkx=3.3 - numpy=1.26.4 - openssl=3.3.1 - packaging=24.1 - pandas=2.2.2 - parso=0.8.4 - pexpect=4.9.0 - pickleshare=0.7.5 - pip=24.0 - platformdirs=4.2.2 - prompt-toolkit=3.0.47 - psutil=5.9.8 - ptyprocess=0.7.0 - pure_eval=0.2.2 - pygments=2.18.0 - python=3.10.8 - python-dateutil=2.9.0 - python-tzdata=2024.1 - python_abi=3.10 - pytorch=2.3.1 - pytz=2024.1 - pyyaml=6.0.1 - pyzmq=26.0.3 - readline=8.2 - setuptools=70.0.0 - six=1.16.0 - stack_data=0.6.2 - sympy=1.12 - tk=8.6.13 - tornado=6.4.1 - tqdm=4.66.4 - traitlets=5.14.3 - typing_extensions=4.12.2 -

tzdata=2024a - wcwidth=0.2.13 - wheel=0.43.0 - xz=5.2.6 - yaml=0.2.5 - zeromq=4.3.5 - zipp=3.19.2 - pip: - aiohttp==3.9.5 - aiosignal==1.3.1 - alabaster==0.7.16 - async-timeout==4.0.3 - attrs==23.2.0 - babel==2.15.0 - blinker==1.8.2 - certifi==2024.6.2 - charset-normalizer==3.3.2 - chemprop==1.6.1 - click==8.1.7 - cloudpickle==3.0.0 - contourpy==1.2.1 - cycler==0.12.1 - docstring-parser==0.16 - docutils==0.21.2 - flask==3.0.3 - fonttools==4.53.0 - frozenlist==1.4.1 - fsspec==2024.6.0 - future==1.0.0 - hyperopt==0.2.7 - idna==3.7 - imagesize==1.4.1 - itsdangerous==2.2.0 - joblib==1.4.2 - kiwisolver==1.4.5 - matplotlib==3.9.0 - multidict==6.0.5 - mypy-extensions==1.0.0 - pandas-flavor==0.6.0 - pillow==10.3.0 - protobuf==3.20.1 - py4j==0.10.9.7 - pyparsing==3.1.2 - rdkit==2023.9.6 - rdkit-pypi==2022.9.5 - requests==2.32.3 - scikit-learn==1.5.0 - scipy==1.13.1 - simsimd==3.7.7 - snowballstemmer==2.2.0 - sphinx==7.3.7 - sphinxcontrib-applehelp==1.0.8 - sphinxcontrib-devhelp==1.0.6 - sphinxcontrib-htmlhelp==2.0.5 - sphinxcontrib-jsmath==1.0.1 - sphinxcontrib-qthelp==1.0.7 - sphinxcontrib-serializinghtml==1.1.10 - tensorboardx==2.6.2.2 - threadpoolctl==3.5.0 - tomli==2.0.1 - torch-geometric==2.5.3 - torch-summary==1.4.5 - typed-argument-parser==1.10.0 - typing-inspect==0.9.0 - urllib3==2.2.1 - werkzeug==3.0.3 - xarray==2024.6.0 - yarl==1.9.4

Additionally, a seperate environment with the following packages is used for Chemprop representation of SMILEs:
- appnope=0.1.4 - asttokens=2.4.1 - backcall=0.2.0 - bzip2=1.0.8 - ca-certificates=2024.6.2 - comm=0.2.2 - debugpy=1.8.1 - decorator=5.1.1 - executing=2.0.1 - importlib_metadata=7.1.0 - ipykernel=6.29.4 - ipython=8.12.2 - jedi=0.19.1 - jupyter_client=8.6.2 - jupyter_core=5.7.2 - krb5=1.21.2 - libcxx=17.0.6 - libedit=3.1.20191231 - libffi=3.4.2 - libsodium=1.0.18 - libsqlite=3.44.2 - libzlib=1.2.13 - matplotlib-inline=0.1.7 - ncurses=6.4 - nest-asyncio=1.6.0 - openssl=3.3.1 - parso=0.8.4 - pexpect=4.9.0 - pickleshare=0.7.5 - pip=23.3.2 - platformdirs=4.2.2 - prompt-toolkit=3.0.47 - prompt_toolkit=3.0.47 - psutil=5.9.8 - ptyprocess=0.7.0 - pure_eval=0.2.2 - python=3.8.18 - python_abi=3.8 - pyzmq=26.0.3 - readline=8.2 - setuptools=69.0.3 - six=1.16.0 - stack_data=0.6.2 - tk=8.6.13 - tornado=6.4.1 - traitlets=5.14.3 - typing_extensions=4.12.2 - wcwidth=0.2.13 - wheel=0.42.0 - xz=5.2.6 - zeromq=4.3.5 - pip: - aiohttp==3.9.5 - aiosignal==1.3.1 - alabaster==0.7.13 - async-timeout==4.0.3 - attrs==23.2.0 - babel==2.14.0 - blinker==1.7.0 - certifi==2023.11.17 - charset-normalizer==3.3.2 - chemprop==1.6.1 - click==8.1.7 - cloudpickle==3.0.0 - contourpy==1.1.1 - cycler==0.12.1 - descriptastorus==2.6.1 - docstring-parser==0.15 - docutils==0.20.1 - filelock==3.13.1 - flask==3.0.0 - fonttools==4.47.2 - frozenlist==1.4.1 - fsspec==2023.12.2 - future==0.18.3 - hyperopt==0.2.7 - idna==3.6 - imagesize==1.4.1 - importlib-metadata==7.0.1 - importlib-resources==6.1.1 - itsdangerous==2.1.2 - jinja2==3.1.3 - joblib==1.3.2 - kiwisolver==1.4.5 - markupsafe==2.1.3 - matplotlib==3.7.4 - mpmath==1.3.0 - multidict==6.0.5 - mypy-extensions==1.0.0 - networkx==3.1 - numpy==1.24.4 - packaging==23.2 - pandas==2.0.3 - pandas-flavor==0.6.0 - pillow==10.2.0 - protobuf==4.25.2 - py4j==0.10.9.7 - pygments==2.17.2 - pyparsing==3.1.1 - python-dateutil==2.8.2 - pytz==2023.3.post1 - rdkit==2023.9.4 - requests==2.31.0 - scikit-learn==1.3.2 - scipy==1.10.1 - simsimd==3.7.7 - snowballstemmer==2.2.0 - sphinx==7.1.2 - sphinxcontrib-applehelp==1.0.4 - sphinxcontrib-devhelp==1.0.2 - sphinxcontrib-htmlhelp==2.0.1 - sphinxcontrib-jsmath==1.0.1 - sphinxcontrib-qthelp==1.0.3 - sphinxcontrib-serializinghtml==1.1.5 - sympy==1.12 - tensorboardx==2.6.2.2 - threadpoolctl==3.2.0 - torch==2.1.2 - torch-geometric==2.5.3 - torchsummary==1.5.1 - tqdm==4.66.1 - typed-argument-parser==1.9.0 - typing-extensions==4.9.0 - typing-inspect==0.9.0 - tzdata==2023.4 - urllib3==2.1.0 - werkzeug==3.0.1 - xarray==2023.1.0 - yarl==1.9.4 - zipp==3.17.0

For manuscripts utilizing custom algorithms or software that are central to the research but not yet described in published literature, software must be made available to editors and reviewers. We strongly encourage code deposition in a community repository (e.g. GitHub). See the Nature Portfolio guidelines for submitting code & software for further information.

Data

Policy information about availability of data

All manuscripts must include a data availability statement. This statement should provide the following information, where applicable:

- Accession codes, unique identifiers, or web links for publicly available datasets
- A description of any restrictions on data availability
- For clinical datasets or third party data, please ensure that the statement adheres to our policy

Data relating to financial estimations regarding chemical screening in yeast is made available in supplementary file S1. The ChEMBL data used in this study is from http://www.ebi.ac.uk/chembl the version of ChEMBL is ChEMBL07 and used under license CC Attribution-ShareAlike 3.0 Unported license. The IUPHAR/BPS data is from https://www.guidetopharmacology.org under CC Attribution-ShareAlike 4.0 International License. The raw subsets of both datasets and processed and combined versions are available in the Zenodo database CSNN with DOI 10.5281/zenodo.12532113 under Creative Commons Attribution 4.0 International license. Source data for graphs are provided in the supplementary source data file.

Research involving human participants, their data, or biological material

Policy information about studies with human participants or human data. See also policy information about sex, gender (identity/presentation), and sexual orientation and race, ethnicity and racism.

Reporting on sex and gender	No human participants, data or biological material
Reporting on race, ethnicity, or other socially relevant groupings	No human participants, data or biological material
Population characteristics	No human participants, data or biological material
Recruitment	No human participants, data or biological material
Ethics oversight	No human participants, data or biological material

Note that full information on the approval of the study protocol must also be provided in the manuscript.

Field-specific reporting

Please select the one below that is the best fit for your research. If you are not sure, read the appropriate sections before making your selection.

☒ Life sciences ☐ Behavioural & social sciences ☐ Ecological, evolutionary & environmental sciences

For a reference copy of the document with all sections, see nature.com/documents/nr-reporting-summary-flat.pdf

Life sciences study design

All studies must disclose on these points even when the disclosure is negative.

Sample size	the sample size of 539 compounds were the maximum number of compounds we could procure on our budget, and was enough samples that we could reliably use population statistics (Z-scores) to determine hit/no hit independently on each receptor chosen. (see methods)
Data exclusions	we excluded 2 compounds from the CHEMBL database for which there was no reported structure or name, which made it impossible to embed in our chemical space neighborhoods which rely on chemical fingerprints calculated from compound structures.
Replication	Our screen was directly compared to prior art from ChEMBL and IUPHAR/BPS, showing good reproducibility. In cases where there is disagreement, we describe so in depth in the supplementary text. ML models we trained as independent replicates on the same splits for the iid and stratified setting. All model weights, processed data, and inference scripts are made a available to replicate figures and data.
Randomization	The prestwick library was randomly distributed over 5 96 well plates, and the chemfaces chemical library on another 2 96 well plates. these layouts were maintained throughout the study.
Blinding	All yeast screening results were withheld from ML model development as not to bias results. The ML model development and optimization was carried out independently.

Reporting for specific materials, systems and methods

We require information from authors about some types of materials, experimental systems and methods used in many studies. Here, indicate whether each material, system or method listed is relevant to your study. If you are not sure if a list item applies to your research, read the appropriate section before selecting a response.

Materials & experimental systems

n/a	Involved in the study	
☒	☐	Antibodies
☒	☐	Eukaryotic cell lines
☒	☐	Palaeontology and archaeology
☒	☐	Animals and other organisms
☒	☐	Clinical data
☒	☐	Dual use research of concern
☒	☐	Plants

Methods

n/a	Involved in the study	
☒	☐	ChIP-seq
☒	☐	Flow cytometry
☒	☐	MRI-based neuroimaging

Plants

Seed stocks	*Report on the source of all seed stocks or other plant material used. If applicable, state the seed stock centre and catalogue number. If plant specimens were collected from the field, describe the collection location, date and sampling procedures.*
Novel plant genotypes	*Describe the methods by which all novel plant genotypes were produced. This includes those generated by transgenic approaches, gene editing, chemical/radiation-based mutagenesis and hybridization. For transgenic lines, describe the transformation method, the number of independent lines analyzed and the generation upon which experiments were performed. For gene-edited lines, describe the editor used, the endogenous sequence targeted for editing, the targeting guide RNA sequence (if applicable) and how the editor was applied.*
Authentication	*Describe any authentication procedures for each seed stock used or novel genotype generated. Describe any experiments used to assess the effect of a mutation and, where applicable, how potential secondary effects (e.g. second site T-DNA insertions, mosiacism, off-target gene editing) were examined.*
